# Coupling of Perinuclear Actin Cap and Nuclear Mechanics in Regulating Flow‐Induced Yap Spatiotemporal Nucleocytoplasmic Transport

**DOI:** 10.1002/advs.202305867

**Published:** 2023-12-31

**Authors:** Tianxiang Ma, Xiao Liu, Haoran Su, Qiusheng Shi, Yuan He, Fan Wu, Chenxing Gao, Kexin Li, Zhuqing Liang, Dongrui Zhang, Xing Zhang, Ke Hu, Shangyu Li, Li Wang, Min Wang, Shuhua Yue, Weili Hong, Xun Chen, Jing Zhang, Lisha Zheng, Xiaoyan Deng, Pu Wang, Yubo Fan

**Affiliations:** ^1^ Key Laboratory of Biomechanics and Mechanobiology (Beihang University), Ministry of Education, Beijing Advanced Innovation Center for Biomedical Engineering, School of Biological Science and Medical Engineering Beihang University Beijing 100083 China; ^2^ Biomedical Pioneering Innovation Center (BIOPIC) Peking University Beijing 100871 China; ^3^ Academy for Advanced Interdisciplinary Studies Peking University Beijing 100871 China; ^4^ Department of Gynecology and Obstetrics Strategic Support Force Medical Center Beijing 100101 China; ^5^ School of Engineering Medicine Beihang University Beijing 100083 China

**Keywords:** flow shear stress, mechanochemical modeling, mechanotransduction, perinuclear actin cap, YAP

## Abstract

Mechanical forces, including flow shear stress, govern fundamental cellular processes by modulating nucleocytoplasmic transport of transcription factors like Yes‐associated Protein (YAP). However, the underlying mechanical mechanism remains elusive. In this study, it is reported that unidirectional flow induces biphasic YAP transport with initial nuclear import, followed by nuclear export as actin cap formation and nuclear stiffening. Conversely, pathological oscillatory flow induces slight actin cap formation, nuclear softening, and sustained YAP nuclear localization. To elucidate the disparately YAP spatiotemporal distribution, a 3D mechanochemical model is developed, which integrates flow sensing, cytoskeleton organization, nucleus mechanotransduction, and YAP transport. The results unveiled that despite the significant localized nuclear stress imposed by the actin cap, its inherent stiffness counteracts the dispersed contractile stress exerted by conventional fibers on the nuclear membrane. Moreover, alterations in nuclear stiffness synergistically regulate nuclear deformation, thereby governing YAP transport. Furthermore, by expanding the single‐cell model to a collective vertex framework, it is revealed that the irregularities in actin cap formation within individual cells have the potential to induce topological defects and spatially heterogeneous YAP distribution in the cellular monolayer. This work unveils a unified mechanism of flow‐induced nucleocytoplasmic transport, providing a linkage between transcription factor localization and mechanical stimulation.

## Graphical abstract

This study unveils a mechanism where flow shear stress modulates Yes‐associated Protein (YAP) nucleocytoplasmic transport. A novel 3D mechanochemical model is developed, integrating flow sensing, cytoskeleton organization, and nucleus mechanotransduction. Results show how actin cap formation and nuclear stiffness alterations influence YAP transport, revealing a unified mechanism of flow‐induced transcription factors transport and its implications on cellular monolayers.

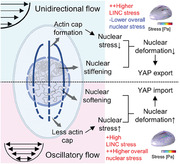

## 1 Introduction

Living cells are constantly subjected to distinct mechanical cues, such as matrix viscoelasticity,^[^ ^1^ ^]^ compressive forces,^[^ ^2^ ^]^ and flow shear stress.^[^ ^3^ ^]^ These forces are transmitted to the nucleus through the cytoskeleton, causing nuclear deformation and regulating the nucleocytoplasmic transport of transcription factors in a mechanosensitive manner,^[^ ^4^ ^]^ such as Yes‐associated Protein (YAP).^[^ ^5^ ^]^ Flow shear stress is particularly relevant for cells in contact with fluids like blood, lymphatic, and interstitial flow, profoundly influencing cellular processes and diseases, such as atherosclerosis and cancer.^[^ ^6^ ^]^ Although chemical pathways, such as Hippo signaling and angiomotin (AMOT) pathways, governing flow‐dependent YAP distribution have been identified under various flow conditions,^[^ ^7^ ^]^ the mechanical mechanism how flow induces the YAP nucleocytoplasmic transport remains largely unclear.

Flow shear stress can induce perinuclear actin cap formation.^[^ ^8^ ^]^ This specialized actin structure is a highly organized, dynamic, oriented, thick cables that tightly cover the apical surface of nucleus through the linker of nucleoskeleton and cytoskeleton (LINC) complexes.^[^ ^9^ ^]^ Notably, the actin cap exerts higher tension compared to conventional fibers and plays a crucial role in YAP‐associated mechanotransduction.^[^ ^10^ ^]^ Additionally, nuclear stiffness itself affects nuclear deformability.^[^ ^2^, ^4^, ^11^ ^]^ Based on these clues, we hypothesize that the YAP nucleocytoplasmic shuttling under different flow conditions is regulated by the nuclear deformation, which is controlled by the actin reorganization and nuclear mechanics alteration. We combined microfluidic vascular chip and Brillouin microscopy, and found that unidirectional flow induces biphasic YAP nucleocytoplasmic transport with initial YAP nuclear import, followed by nuclear export as actin cap formation and nuclear stiffening occur. In contrast, oscillatory flow maintains YAP nuclear localization through slight actin cap formation and nuclear softening. We then developed a mechanochemical model accounting for flow sensing at the plasma membrane, subsequent effects on the cytoskeleton, force transmission to the nucleus, and impact on YAP transport. Our findings reveal that actin cap concentrates stress on linkers of nucleus to cytoskeleton (LINC) complex while reducing overall nuclear membrane stress exerted by conventional fibers. The increasing nuclear stiffness collaboratively inhibit nuclear membrane deformation, ultimately governing the biphasic nucleocytoplasmic transport of YAP. Furthermore, we extend this single‐cell framework – in a minimal way – to the analysis of cell monolayer, and predict that irregularities in actin cap formation within individual cells dictate the collective topological defects and spatially heterogeneous YAP distribution in monolayer.

## 2 Results

### 2.1 Flow‐Induced Actin Cap Formation and Nuclear Stiffness Alterations are Associated with YAP Nucleocytoplasmic Transport

Expanding on previous investigations of endothelial cell morphological response to flow,^[^ ^12^ ^]^ we examined the distinct effects of unidirectional and oscillatory shear stress on actin cap formation, nuclear stiffness, and YAP nucleocytoplasmic distribution using a microfluidic system as shown in Figure S1a–c (Supporting Information). With this system, we ensured that the cell density and cell‐cell contact remained stable under flow condition (Figure S1d,e, Movies S1 and S2, Supporting Information). In addition, cells exhibited stability when cultured statically for 24 h without flow stimulation (Figure S1f, Supporting Information).

**Figure 1:**
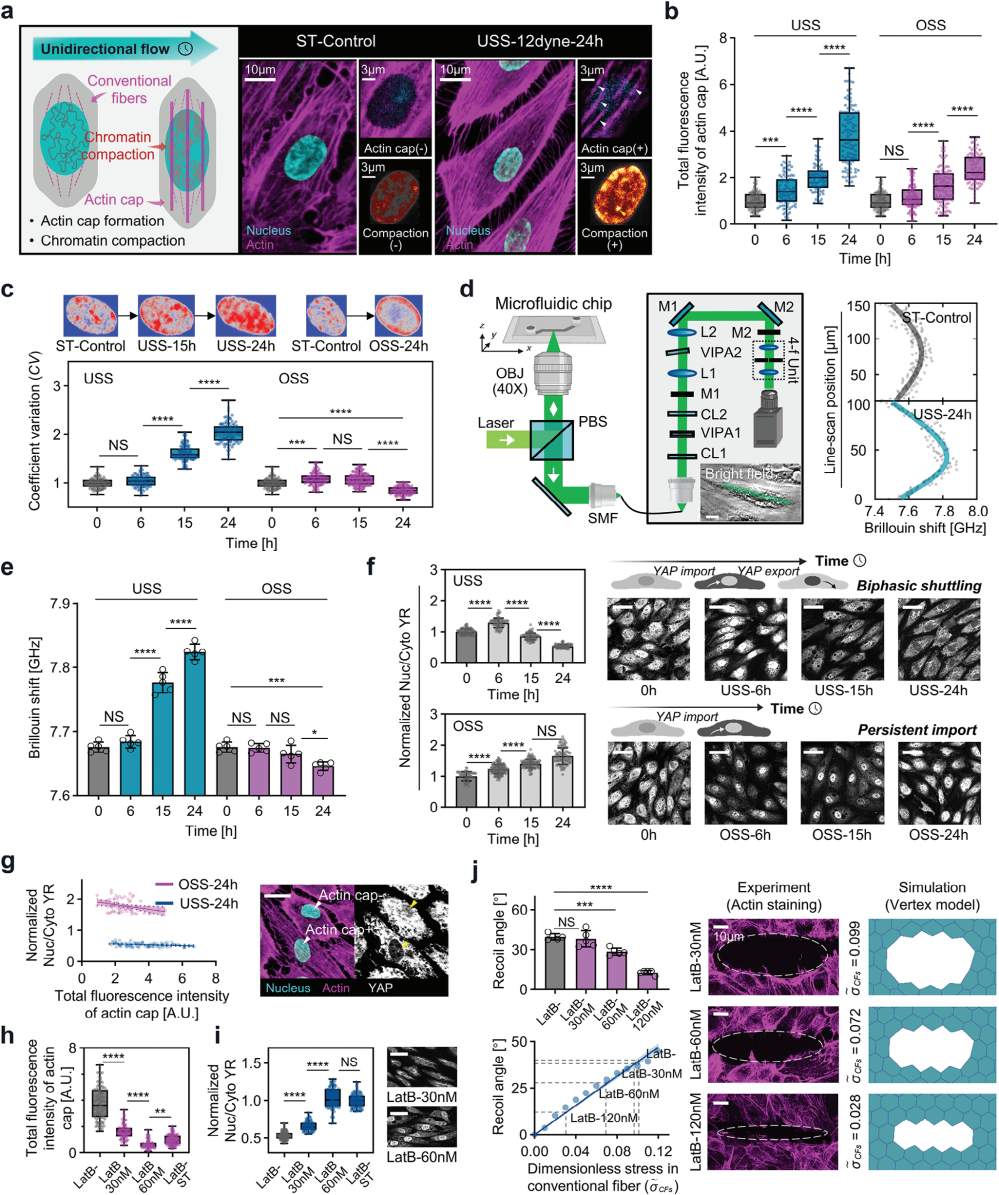
The connection between actin cap formation, nuclear mechanics alteration, and YAP localization under flow shear stress. a) Schematic of endothelial cell subjected to unidirectional flow with increasing actin cap formation and chromatin compaction. Arrowhead indicates the actin cap and chromatin compaction. b,c) Total fluorescence intensity of actin cap and coefficient of variation (CV) in response to unidirectional shear stress (USS, 12 dyne cm^−2^) and oscillatory shear stress (OSS, 0±12 dyne cm^−2^, 1 Hz) (3 independent experiments, n > 80 cells, normalized to mean of control). d) Brillouin microscopy combined with the microfluidic chip (Scale bars, 20 µm). e) Time‐dependent increase in Brillouin shift in response to USS and OSS (n = 5 for each condition). f) Cells subjected to USS with YAP showing initial nuclear localization followed by cytoplasmic retention, and cells subjected to OSS with continuous YAP nuclear localization (IMARIS easy 3D projection, Scale bars, 40 µm). Quantification of YAP nuclear‐cytoplasmic ratio (YR) in response to USS and OSS (3 independent experiments, n > 80 cells, normalized to mean of control). g) Linear regression of normalized YR and total fluorescence intensity of actin cap under 24 h USS and OSS (p < 0.01). The schematic diagram illustrates that cells with actin cap exhibit a lower YR (Scale bars, 20 µm). h,i) Total fluorescence intensity of actin cap and normalized YR of LatB‐untreated, 30, and 60 nM under 24 h USS (12 dyne cm^−2^), and the static control (3 independent experiments, n > 80 cells. Scale bars, 20 µm). j) Experimental recoil angle results following laser ablation under various doses of LatB (top left, n = 5 for each condition); Vertex simulation shows a positive correlation between recoil angle and dimensionless stress within conventional fibers (bottom left, dash lines indicate the experimental measured recoil angle and the corresponding stress); Schematic of laser ablation experiment with 30, 60, and 120 nM LatB, and the corresponding vertex simulation (right panel). All data are shown as mean ± s.d. *p<0.05; **p<0.01; ***p<0.001; ****p<0.0001; NS, not significant.

The percentage of cells with actin cap (Figure S2a,b, Supporting Information), the total fluorescence intensity of actin cap and the chromatin compaction increased with flow in a time‐ and magnitude‐dependent manner under both 12 dyne cm^−2^ (**Figure** **1**a–c) and 4 dyne cm^−2^ unidirectional shear stress (Figure S3a–c, Supporting Information). To further validate the formation of actin cap, we performed Nesprin 1 staining,^[^ ^13^ ^]^ a component of the LINC complex, and also observed a gradual increase in the frequency of Nesprin line‐positive nuclei with prolonged exposure to unidirectional shear stress (Figure S2c, Supporting Information). Considering the connection between nuclear stiffness and chromatin compaction,^[^ ^14^ ^]^ we employed Brillouin microscopy for label‐free and contact‐free measurements of nuclear stiffness (Figure 1d; Figure S4a, Supporting Information). The results show the increased nuclear stiffness under both unidirectional conditions with flow time (Figure 1e; Figure S3d, Supporting Information). Complementing this, we also noted an increase in cytoplasmic stiffness due to unidirectional flow (Figure S4b, Supporting Information) and an augmented fluorescence intensity of H3K9me3 in the nucleus (Figure S2d, Supporting Information), which aligns with the recent study suggesting that H3K9me3‐marked heterochromatin contributes to nuclear stiffening.^[^ ^15^ ^]^ Furthermore, YAP initially entered and subsequently gradually exported from the nucleus (Figure 1f; Figure S3e, Supporting Information), in line with previous studies.^[^ ^7a^ ^]^ In contrast, oscillatory shearing (1 Hz, 0 ± 12 dyne cm^−2^) resulted in more gradual and less extensive actin cap formation (Figure 1b), with slightly reduced chromatin compaction and nuclear stiffness (Figure 1c and e). Notably, YAP remained localized in the nucleus under the oscillatory shear stress (Figure 1f), corroborating prior findings.^[^ ^7b^ ^]^

We furthermore conducted correlation matrix analysis among the mean YAP nuclear‐cytoplasmic ratio (YR), actin cap, and nuclear stiffness based on the above statistical results. We identified a significant negative correlation between YR and nuclear stiffness (Figure S3f, Supporting Information). Additionally, we observed the negative correlation between YR and the total fluorescence intensity of the actin cap (P<0.05). These findings deviate from the conventional understanding of the actin cap's role in promoting YAP nuclear accumulation under basal stretch instead of flow shear stress.^[^ ^10^ ^]^ To delve deeper into the contributions of the actin cap in flow‐induced mechanotransduction, we examined the relationship between the total fluorescence intensity of actin cap and YR in individual cells under different flow conditions. Our results demonstrated that cells with higher actin cap intensity exhibited lower YR values, both in the presence of unidirectional and oscillatory shear stress (Figure 1g; Figure S3g, Supporting Information). In addition, to experimentally validate the role of actin cap, we suppressed its formation using low doses of latrunculin B (LatB, <60 nM) under 12 dyne cm^−2^ unidirectional shear stress, as described in previous studies.^[^ ^9^, ^16^ ^]^ Upon inhibiting (Figure 1h; Figure S5a, Supporting Information), a significant increase in YR was observed after 24 h of shearing compared to the group without actin cap inhibition (Figure 1i).

To better address potential off‐target effects of LatB on other actin structures, we first looked at the structural integrity of conventional fibers at varying concentrations of LatB through immunofluorescence staining. Our findings revealed that at concentrations below 60 nM, LatB effectively maintained the structural integrity of conventional fibers, exhibiting only marginal changes in the fluorescence intensity (Figure S5b, Supporting Information). The dose effects of LatB on conventional fibers’ contractile function was further examined by laser ablation experiments and simulations. As illustrated in Figure 1j (top left), 30 nM LatB treatment has no significant impact on the recoil angle, which was calculated as the ratio of the short axis to the long axis of the opening ellipse, while a 60 nM dose results in a slight reduction. Subsequent simulation using a vertex model (detailed in Supporting Information, Section 4) illustrate a linear correlation between recoil angle and contractile stress (Figure 1j, bottom left). Consequently, the experimental observed alterations in recoil angles at 30 and 60 nM LatB treatments correlates with slight reductions in contractile stress, quantified at less than 9% and 36%, respectively (Figure 1j, right). In contrast, the 120 nM concentration of LatB markedly diminished contractile stress, reinforcing our selection of sub‐60 nM levels for experimentation. These results are consistent with previous studies suggesting that low‐concentration LatB preferentially inhibits actin cap formation, as conventional fibers possess a more accessible actin pool, while actin cap synthesis requires additional resources from these fibers.^[^ ^9^, ^16^ ^]^

### 2.2 Mechanochemical Model to Analyze the Flow Shear Stress Transmitted to Nucleus Regulating Nucleocytoplasmic Transport of YAP

**Figure 2:**
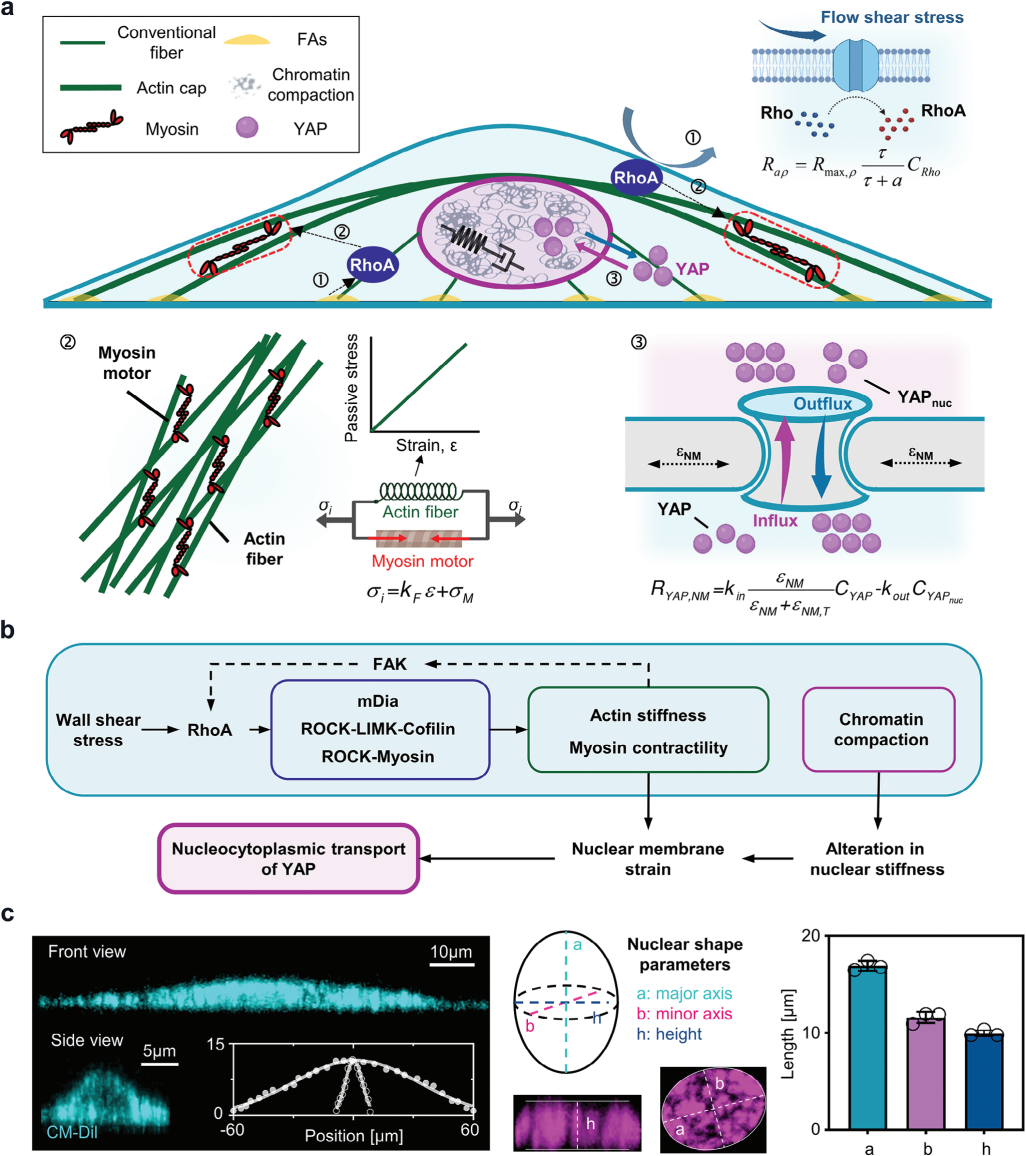
The development of 3D mechanochemical model. a) The components considered in the model, including the cytoplasm, conventional fiber, actin cap, nucleus, and biochemical pathways centered on RhoA to regulate the transport of YAP. b) Biochemical pathways involved in the model. c) Geometric parameters obtained from cytoplasm (stained with CM‐Dil), fitted by the Gaussian curve, and nuclear images (stained with DAPI), described by the length of major axis, minor axis, and height.

A 3D cell is included in the model, which comprises the cytoplasm, conventional fibers, actin cap, nucleus (**Figure** **2**a; Figure S6a, Supporting Information), and mechanotransductive pathways upstream to YAP (Figure 2b). The deformable cell is embedded in a microfluidic channel and subjected to flow (Figure S6b, Supporting Information). Using immunofluorescence staining with CM‐Dil, phalloidin, and DAPI, the model effectively captures these subcellular components (Figure 2c; see Figure S7a, Supporting Information and Section 1). We considered both the involved biochemical pathways and mechanical transmission, including the activation of RhoA (numbered as ① in Figure 2a), cytoskeleton reorganization (numbered as ② in Figure 2a), and YAP nucleocytoplasmic transport (numbered as ③ in Figure 2a) in this model.

#### 2.2.1 Flow‐Induced RhoA Activation

First, flow shear stress activates integrins and G protein‐coupled receptors at plasma membrane, leading to RhoA activation,^[^ ^17^ ^]^ expressed by Hill equation:

(1) $$R_{a\rho }=R_{\max,\rho }\;\frac{\tau }{\tau +a}C_{Rho}$$

Here, *R_max,ρ_* represents the maximum activation rate, *τ* denotes shear stress magnitude, *C_Rho_* is Rho concentration, and *a* is shear stress value when the activation rate of RhoA reaches *R_max,ρ_*/2. Additionally, stress within actin fibers at the focal adhesion would lead to the phosphorylation of FAK and further activate RhoA.^[^ ^18^ ^]^

#### 2.2.2 Cytoskeleton Reorganization

Following RhoA activation, rho‐associated kinases (ROCK), formin mDia1 (mDia), LIM kinase (LIMK) and Cofilin pathways are activated (Figure 2b). The transporting processes of these components are governed by reaction‐diffusion equations (see Supporting Information, Section 2.4), with coefficients obtained from recent biochemical models.^[^ ^18^, ^19^ ^]^ These pathways intricately regulate the concentrations of F‐actin and activated myosin, collectively governing the cytoskeletal stress. Specifically, the cytoskeletal stress arises from the interplay of active and passive constituents, where *σ_i_* = *K_F_ε*+*σ_M_*. Here, *K_F_* represents the passive cytoskeletal stiffness, and *σ_M_* denotes the myosin‐driven contractile stress.^[^ ^11^, ^20^ ^]^ For the conventional fibers, we incorporated their dynamic reorganization by modeling the volume fraction as a first‐order function dependent on the concentrations of F‐actin (see Supporting Information, Section 2.4). Additionally, to capture the anisotropic characteristics of the conventional fiber, we incorporated its directionality, aligned with the diffusion potential of F‐actin from the basal surface of the cell toward the nuclear membrane (see Figure S6c, Supporting Information, and Section 2.3 for more details). Regarding the actin cap, its formation mechanism in response to flow shear stress is complex. As illuminated by the seminal research of Chambliss et al.,^[^ ^21^ ^]^ flow‐induced actin cap formation is governed by zyxin at low shear stress and by talin at high shear stress. Thus, we take no account of the formation process of actin cap structure and incorporated the formed structure of actin cap with various volume fraction based on the experimental measurements under each flow scenarios (Figure S7b, Supporting Information).

#### 2.2.3 YAP Nucleocytoplasmic Transport

Lastly, we modeled a set of reactions accounting for the interplay between F‐actin, myosin, and YAP dephosphorylation. The rate of YAP dephosphorylation are modeled as a product of F‐actin and myosin concentration,^[^ ^18a^ ^]^ while YAP nuclear localization or cytoplasmic retention is determined by the export and import rates of dephosphorylated YAP.^[^ ^4b^ ^]^ Specifically, the YAP nuclear import rate is set as a Hill function of nuclear membrane equivalent strain, reflecting nuclear pore stretch, while the YAP export rate follows a first‐order rate^[^ ^4^, ^18^, ^19^ ^]^ (see Supporting Information, Section 2.4). Similar to the method used for fitting the actin cap volume fraction, we utilized Brillouin data as shown in Figure 1e to fit the time course of nuclear stiffness in each flow condition (Figure S7c, Supporting Information).

**Figure 3:**
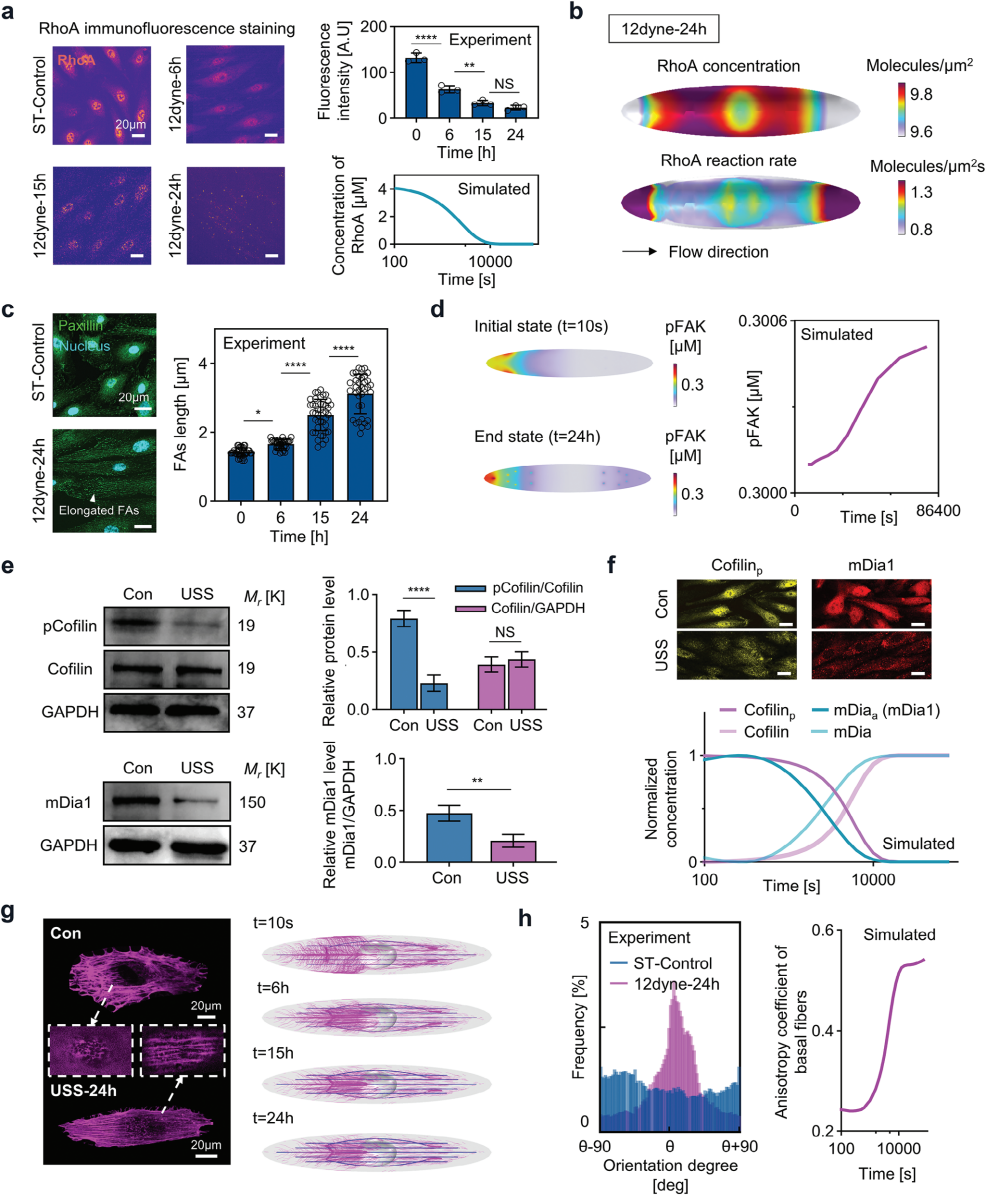
Experimental validations of mechanochemical behaviors involved in shear stress mechanotransduction. a) Comparison between the experimental measured RhoA intensity and model‐simulated RhoA concentration in the cytoplasm. b) Spatial distribution of RhoA concentration and reaction rate at the cell membrane under unidirectional shear stress. c) Experimentally measured focal adhesions length (including both actin cap and conventional fiber associated focal adhesions, n > 30). d) Model‐simulated pFAK concentration (left panel) and the time‐course of mean pFAK concentration on basal surface. e) top left: Phosphorylated and total cofilin expression on static or unidirectional flow (USS), top right: pCofilin/total Cofilin and Cofilin/GAPDH intensity ratio (n > 3), left bottom: mDia1 expression on static or unidirectional flow (USS), right bottom: mDia1/GAPDH intensity ratio (n > 3). f) The comparison between the model‐simulated pCofilin and activated mDia concentration and immunofluorescence imaging (Scale bars, 20 µm). g) Immunofluorescence imaging (basal plane, dashed box indicates the apical plane) and simulated (conventional fiber is colored by magenta and actin cap is colored by blue) actin fibers formation. The density of the streamline presented in the simulated results is determined by the fibers volume fraction (*ρ*). h) Experimental and simulated polarization of conventional fibers, described by the anisotropy coefficient calculated as the ratio of stress parallel to flow versus perpendicular to flow.

To validate the accuracy of the proposed mechanochemical model, we compared the model predictions with experiments under 12 dyne cm^−2^ conditions. Our initial findings demonstrated a notable agreement between the model‐predicted RhoA concentrations and those discerned via immunofluorescence staining and previously documented data,^[^ ^17^ ^]^ demonstrating a decline in RhoA activity with extended durations of unidirectional flow (**Figure** **3**a and b). Furthermore, immunofluorescence imaging revealed an increase in the mean length of focal adhesions (averaged across conventional fiber and actin cap associated focal adhesions) as unidirectional flow was applied (Figure 3c). This trend aligns with our model's prediction about the average concentration of phosphorylated FAK in the cellular basal plane (Figure 3d). To gain deeper insights, we analyzed the retrograde actin flow, which is documented to be associated with FA elongation.^[^ ^22^ ^]^ Utilizing Lifeact‐mCherry for real‐time visualization of F‐actin (detailed in Methods^[^ ^23^ ^]^), we noted a marked reduction in the average speed of retrograde actin flow with sustained unidirectional shear (Figure S8a, Supporting Information). Correlating these findings with our model, we discerned a concomitant decrease in simulated basal plane traction force, an indicator of the retrograde actin flow velocity,^[^ ^24^ ^]^ aligning with the slowdown of F‐actin temporal rates upon shear application (Figure S8b, Supporting Information).

Furthermore, we conducted Western Blot (WB) analyses (see Methods) to examine the levels of phosphorylated and total Cofilin, as well as mDia1, which are key upstream signaling molecules of the cytoskeleton featured in our model. The data reveal that while the total levels of Cofilin remain unchanged, there is a significant decrease in phosphorylated Cofilin when subjected to unidirectional flow. In addition, we observed a downregulation of mDia1 in response to the flow, as shown in Figure 3e. These findings are corroborated by our immunofluorescence staining results for phosphorylated Cofilin and mDia1, detailed in Figure S8c,d (Supporting Information). Correspondingly, our model reflects this trend, indicating a decrease in phosphorylated Cofilin and a reduction in activated mDia1, which is depicted in Figure 3f.

Mechanistically, our model successfully captures the conventional fiber formation and polarization under flow shear stress (Figure 3g,e; Figure S8e, Supporting Information). Additionally, our model aligns well with experimentally measured changes in nuclear height, reflecting the overall nuclear deformation. Specifically, we observed a decrease in nuclear height in response to flow shear stress, followed by a subsequent rebound after 15 h (Figure S8f, Supporting Information). Lastly, our model successfully reproduced correlations among the involved signaling molecules: positive correlations between RhoA and downstream activated ROCK and myosin, positive correlations between RhoA and downstream mDia and F‐actin, and correlations between RhoA, activated ROCK, LIMK, Cofilin, and F‐actin (Figure S8g, Supporting Information).The detailed model development, parameter values, and sensitivity analysis of key parameters are provided in Section 1–3, Tables S1 and S2, and Figure S9b (Supporting Information), respectively.

### 2.3 Combined Effects of Actin Cap Formation and Nuclear Mechanics on Biphasic YAP Nucleocytoplasmic Shuttling under Unidirectional Flow Shear Stress

In an effort to further our understanding of the biphasic pattern of YAP transport, we first integrated actin caps formation and nuclear stiffening data from 12 dyne cm^−2^ unidirectional flow experiments (see Figure S7b,c, Supporting Information) into the model (refers to ACs+, Stiff+ condition).

**Figure 4:**
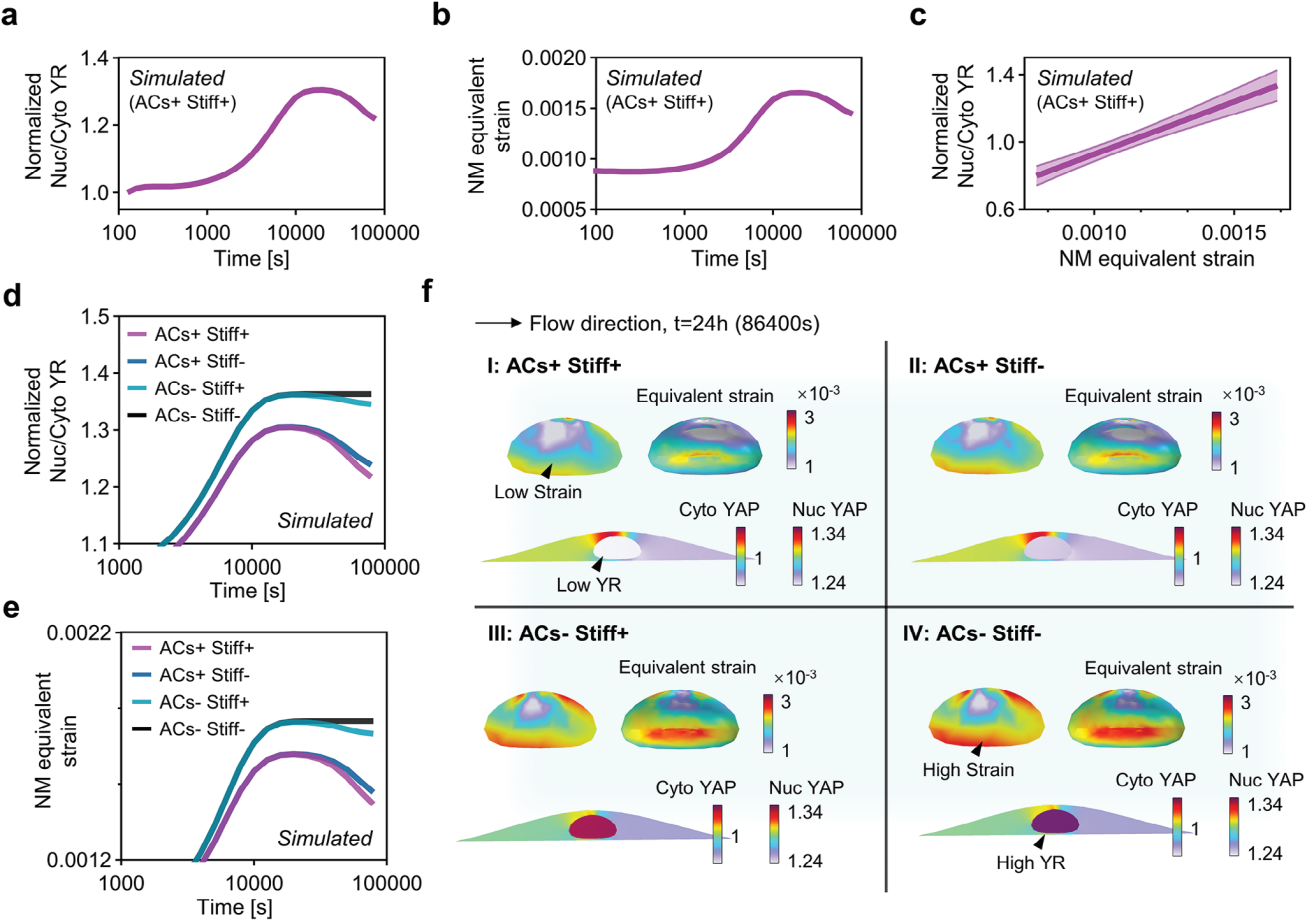
The roles of actin cap and nuclear mechanics in regulating YAP biphasic transport. a,b) Simulated YAP nuclear‐cytoplasmic ratio (YR) and nuclear membrane (NM) over a 24‐hour period under unidirectional shear stress (USS) of 12 dyne cm^−2^ when both actin cap formation and nuclear stiffening are present (ACs+, Stiff+). c) Linear regression analysis of YR with respect to NM equivalent strain (p<0.001) demonstrating a positive correlation between these two parameters, consistent with recent experimental findings. d,e) Individual effects of actin cap and nuclear stiffening in YR and NM equivalent strain, illustrating the dominating effects of actin cap and the collaborating effects of nuclear stiffening on the biphasic transport of YAP. f) YR and nuclear membrane strain contour plots of these conditions at a flow time of 24 h (Quadrant I: ACs+, Stiff+; Quadrant II: ACs+, Stiff‐; Quadrant III: ACs‐, Stiff+; Quadrant IV: ACs‐, Stiff‐).

Our simulations successfully replicated the experimentally observed biphasic pattern of YAP transport (Figure 1f). More specifically, the YAP ratio (YR) exhibited an initial increase, followed by a gradual decline over a 24 h flow period (**Figure** **4**a; Movie S3, Supporting Information). In line with the dynamics of YAP shuttling, our model also predicted a biphasic pattern in nuclear membrane strain (Figure 4b). Notably, we found a positive correlation between YR and nuclear membrane strain (Figure 4c), which aligns with recent experimental findings.^[^ ^4a,b^ ^]^ Furthermore, this parallel biphasic dynamic of YAP and nuclear membrane strain was also evident in simulations conducted at 4 dyne cm^−2^ over a duration of 72 h (Figure S10b, Supporting Information).

To investigate whether the coupling of actin cap formation and nuclear stiffening plays a dominant role in YAP biphasic transport, we selectively inhibited these factors in our model (see Figure S10a, Supporting Information for detailed input on actin cap and nuclear stiffness for each group). Our results predicted that inhibiting either actin cap formation or nuclear stiffening individually would lead to a reduction in YAP export and a decrease in nuclear membrane strain under prolonged flow (ACs+, Stiff‐ and ACs‐, Stiff+ conditions in Figure 4d,e). Remarkably, when both factors were simultaneously suppressed, the decreasing trend of YR and nuclear membrane strain disappeared completely (ACs‐, Stiff‐ condition in Figure 4d,e).

Then, we furnished simulated YR and nuclear membrane strain contour plots of these conditions at a flow time of 24 h (Figure 4f). The graphical representations demonstrate that inhibiting actin cap formation alone (refer to “ACs‐, Stiff+” condition in Quadrant III of Figure 4f) leads to a higher YAP nuclear localization and nuclear membrane strain compared to the reference condition wherein both factors are present (“ACs+, Stiff+” condition in Quadrant I of Figure 4f). Furthermore, suppressing nuclear stiffening alone (refer to “ACs+, Stiff‐” condition in Quadrant II of Figure 4f) yields a similar trend as inhibiting actin cap formation, though less pronounced. Consistent with the time‐course spatial averaged results depicted in Figure 4d,e, when both factors are inhibited simultaneously (“ACs‐, Stiff‐” condition in Quadrant IV of Figure 4f), the nuclear membrane strain increases substantially, facilitating a higher concentration of YAP within the nucleus.

These results suggest that the biphasic response of nuclear membrane strain and YAP to unidirectional shear stress is primarily governed by the coupling effects of actin cap and nuclear mechanics.

### 2.4 Mechanotransduction Processes Underlying Flow‐Induced YAP Nucleocytoplasmic Shuttling

**Figure 5:**
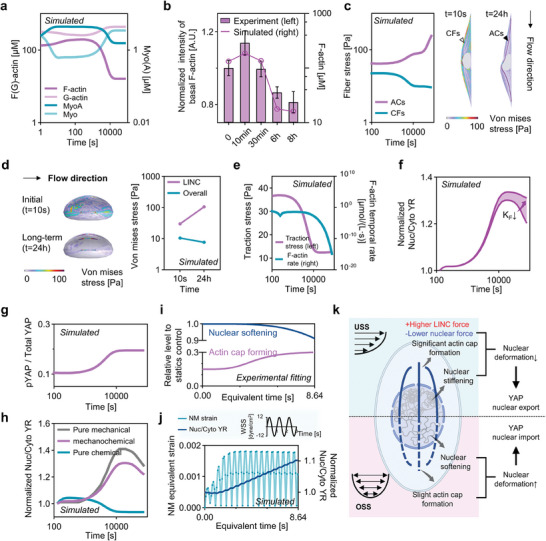
Mechanotransduction processes of flow shear stress and YAP spatiotemporal nucleocytoplasmic shuttling. a) Averaged concentration of F‐actin, G‐actin, myosin, and activated myosin during USS. b) Time course of live‐cell Lifeact‐mCherry intensity at the basal plane (n > 3) and model‐simulated F‐actin mean concentration. c) Averaged Von Mises stress in conventional fibers and actin cap during USS, and the intracellular Von Mises stress distribution inside the cell. The deformation is visualized and expanded by a factor of 100. d) Nuclear stress distribution at the initial (t = 10 s) and end state (t = 24 h) of USS (left), and mean nuclear stress in the actin cap‐associated LINC and nuclear membrane (right). e) Basal averaged traction stress and F‐actin temporal rate decreases as flow prolonged. f) The effect of actin cap stiffness (*K_F_*) on the biphasic response of YAP transport while maintaining its contractile stress. g) The activation of YAP, described by the ratio of pYAP to Total YAP, with respect to USS time. h) The comparison of pure chemical, mechanical, and mechanochemical regulation of YAP transport. i) Relative changes in actin cap and nuclear stiffness under OSS fitted by experimental data. j) NM strain exhibits an oscillatory trend and YR increases as the duration of OSS prolongs. k) Prolonged USS leads to significant formation of actin cap and an increase in nuclear stiffness, while OSS results in slight actin cap formation accompanied by a decrease in nuclear stiffness, collaboratively regulating YAP transport.

To elucidate the mechanisms driving YAP nucleocytoplasmic shuttling in response to flow, we utilized the model to simulate the mechanotransduction process under unidirectional flow of 12 dyne cm^−2^ over 24 h. Initially, our model uncovered a rapid increase in F‐actin and activated myosin concentrations within the conventional fibers, which then gradually decreased (**Figure** **5**a). This pattern was validated by experimental data, specifically the Lifeact‐mCherry intensity measurements from live‐cell imaging (Figure 5b; Figure S10c, Supporting information).

In parallel, the model depicted the stress dynamics within conventional fibers, revealing an initial phase of high stability before a subsequent decrease (Figure 5c; Movie S4, Supporting Information). In contrast, stress levels within actin cap fibers progressively intensified with continuous flow (Figure 5c; Movie S4, Supporting Information). These fiber types collectively modulate nuclear membrane stress. At the initiation of flow, the elevated stress within conventional fibers led to a dispersed stretching exerted on the nucleus (left top of Figure 5d; Movie S5, Supporting Information). With extended flow duration, stress became more concentrated on the LINC protein complex associated with the actin cap (left bottom of Figure 5d; Movie S5, Supporting Information).

We then evaluate the average stress across the nuclear membrane, which determines the nuclear deformation and thus YAP transport. Intriguingly, while stress in the LINC complex notably increased, the overall stress on the nuclear membrane decreased (Figure 5d, right panel). This observation aligns with the deceleration of actin retrograde flow, corroborated both computationally (Figure 5e; Figure S8b, Supporting Information) and experimentally (Figure S8a, Supporting Information). The interplay of reduced nuclear stress and flow‐induced nuclear stiffening culminates in decreased nuclear deformation, thereby favoring YAP export from the nucleus.

Further, our model suggests that the stiffness of the actin cap, in addition to its stress, is instrumental in safeguarding against nuclear deformation. By numerically decreasing the actin cap's stiffness while maintaining its contractile stress, we observed the long‐term decrease in YAP export are inhibited (Figure 5f).

In addition to mechanical regulation, the chemical activity of YAP has been demonstrated to contribute to its transport process.^[^ ^7b,c^ ^]^ Our model results indicate that the ratio of phosphorylated YAP (pYAP) to total YAP (summation of pYAP and dephosphorylated YAP) increases as unidirectional shear stress persists (Figure 5g), consistent with previous experimental studies.^[^ ^7b,c^ ^]^ To elucidate the chemical role of decreased YAP activity in YAP export under long‐term flow, we first suppressed the mechanosensitive aspect of YAP transport in our model. Interestingly, our simulations showed a rapid YAP nuclear export reaching a stable value (Figure 5h), differing from the experimental results on YR dynamics, suggesting mechanical regulation is a crucial mechanism of YAP transport. In addition, when the YAP activity is artificially kept unchanged, the model still demonstrated initial nuclear YAP import followed by export under USS, highlighting mechanical regulation's primacy (Figure 5h).

Extending our analysis to oscillating shear stress, based on the experimental data of actin cap formation and nuclear softening process over 24 h (86 400 s), we condensed it into an 8.64 second process (original experimental fitting data in Figure S7b,c, Supporting Information; shortened data shown in Figure 5i). Our results demonstrate that, due to the highly contractile nature of conventional fibers and the relatively low actin cap formation and nuclear softening compared to unidirectional shear stress, cells experience a significant increase in nuclear membrane strain with each oscillating period. This substantial nuclear membrane deformation consistently promotes YAP nuclear import (Figure 5j). Furthermore, artificially enhancing actin cap formation and inhibiting nuclear softening during oscillating shear stress led to a reduction in both nuclear membrane strain and YAP nuclear entry over time (Figure S10d, Supporting Information).

Building upon the single‐cell model, we then extended our investigation to a collective‐cell framework using the vertex model (see details in Supporting Information Section 4,5). The results indicate that the localized actin cap irregularities can induce topological defects and spatially heterogeneous nucleus membrane strain in cell monolayer (Figure S11, Supporting Information). This suggests a potential influence of actin cap on modulating collective cell behaviors.

## 3 Discussion and Conclusions

Taken together, our study demonstrates how the nucleocytoplasmic shuttling of YAP under flow shear stress is controlled by the coupling between the actin cap and nuclear mechanics. Through the combination of microfluidics and Brillouin microscopy, we identified a negative correlation between YAP nuclear‐cytoplasmic ratio, actin cap, and nuclear stiffness across various flow conditions. Utilizing a comprehensive 3D mechanochemical model, we further elucidated that the formation of actin cap concentrates stress within the LINC complex and provides stiffness constraint to reduce nuclear deformation. Additionally, we revealed that nuclear stiffening decreases the nuclear deformation and, subsequently, regulating YAP nuclear export. Notably, although the nucleocytoplasmic shuttle process of YAP is spatiotemporally varied under various shear stress stimuli, this underlying mechanical mechanism remains consistent. Under prolonged unidirectional flow, the substantial actin cap and nuclear stiffening contribute to reduced nuclear deformation and facilitate YAP nuclear export (Figure 5k), indicating the attainment of physiological homeostasis.^[^ ^7a,c^ ^]^ In contrast, under oscillatory shear stress, the actin cap forms slightly, accompanied by nuclear softening, leading to periodic oscillations in nuclear strain and subsequent YAP nuclear import.^[^ ^7b^ ^]^ This corresponds to various flow‐dependent diseases, such as the localization of atherosclerosis in disturbed flow regions and the enhancement of metastatic cancer cell invasion under oscillatory shear stress.^[^ ^6^ ^]^

Importantly, our proposed mechanochemical model departs from previous models primarily focused on basal stretching and geometric confinements,^[^ ^11^, ^25^ ^]^ which is of significant importance considering the widespread occurrence of flow shear stress experienced by all cells in contact with fluids. Moreover, in contrast to previous YAP models that predominantly based on chemical transport framework,^[^ ^18^, ^19^ ^]^ our model directly couples the chemical transport and mechanical simulation, which allows us to explore the interplay between these two crucial aspects of YAP regulation. In terms of chemical aspects, our results demonstrate that the correlation between the signal molecules in our model and YAP nuclear‐cytoplasmic ratio aligns with the recent chemical model developed by Scott et al.^[^ ^18a^ ^]^ Furthermore, regarding mechanical aspects, our model successfully captures the relationship between nuclear deformation and YAP transport, which is consistent with recent experimental findings indicating that nuclear pore opening facilitates YAP nuclear import.^[^ ^4a,b^ ^]^ Interestingly, our investigation revealed that under unidirectional flow conditions, phosphorylated YAP levels increase, also contributing to YAP export. However, when the YAP's mechanosensitive transport is fully eliminated, YAP rapidly forms cytoplasmic retention, which contradicts the experimentally observed biphasic YAP transport (Figure 5H,I). This suggests that mechanical regulation is a crucial mechanism governing YAP transport.^[^ ^4a,b^ ^]^ In addition, to complement and further validate our model, we addressed the lack of previously published long‐term experimental data on RhoA‐centered pathway under flow. As a result, we performed immunofluorescence staining for RhoA, focal adhesions, mDia, and phosphorylated Cofilin under 24 h of unidirectional flow. Remarkably, the experimental results from our long‐term flow conditions exhibited trends consistent with the published short‐term flow conditions^[^ ^7^, ^17^ ^]^ and closely matched the model predictions.

To validate the role of the actin cap in YAP transport as predicted by the model, we utilized a low dose of LatB to inhibit actin cap formation. To address potential off‐target effects of LatB on other actin structures, we conducted laser ablation experiments to assess the impact of LatB on conventional fibers. Our results indicated that LatB treatment at concentrations below 60 nM had minimal inhibitory effects on conventional fiber contractility and integrity. This finding is consistent with previous studies, as conventional fibers possess a more accessible actin pool, whereas actin cap synthesis requires additional resources from these fibers.^[^ ^9^, ^16^ ^]^ However, it is essential to acknowledge that LatB treatment may still impact other cellular processes, such as calcium activity^[^ ^26^ ^]^ and chromatin compaction alteration.^[^ ^16^ ^]^ Moreover, other studies used inhibitory microRNAs to knock down LINC complexes to suppress actin cap formation. Nonetheless, this approach not only inhibits actin cap but also disrupts the connection and force transmission between conventional fibers and the nucleus, resulting in sustained cytoplasmic retention of YAP.^[^ ^27^ ^]^ Furthermore, LINC knockdown results in the suppression of actin cap formation and concomitant alterations in Lamin A/C expression^[^ ^28^ ^]^ and nuclear integrity.^[^ ^29^ ^]^ Nevertheless, our findings, along with recent experimental results,^[^ ^2b^ ^]^ emphasize that changes in nuclear properties also play a regulatory role in YAP transport. Therefore, we emphasize that conducting experimental validation of the model's predictions is crucial, and subsequently utilizing the model for predictions that are otherwise challenging to investigate experimentally holds significant value.

To assess the nuclear stiffness of cells embedded in microfluidic chips and reduce the impact caused by measurement contact and cell nucleus separation process of the traditional methods, we used a novel optical technique, Brillouin microscopy, capable of measuring nuclear stiffness in a label‐free, non‐contact and non‐invasive manner. To verify the reliability of Brillouin microscopy, we quantified the degree of chromatin compaction and found a strong correlation with the nuclear stiffness, which is consistent with the published well‐proven conventional mechanical measurements.^[^ ^30^ ^]^ In addition, it has been reported that the degree of chromatin compaction is associated with the localization of YAP.^[^ ^31^ ^]^ For example, epithelial cells exposed on convex curvature show an elevated nuclear presence of YAP and less condensed chromatin, while the nucleus on concave curvatures contains more condensed chromatin with cytoplasmic YAP.^[^ ^32^ ^]^ These experimental observations of YAP transport induced by variable mechanical cues might be driven by the alteration of nuclear stiffness and condensed chromatin.

Importantly, although our study focused on YAP, the observed regulatory patterns may have broader implications for other transcription factors, including MRTF‐A, β‐catenin, and MyoD.^[^ ^4a,b^ ^]^ This multiscale framework for comprehending nucleocytoplasmic transport of transcription factors may potentially utilized to identify therapeutic targets to mitigate the detrimental effects of disturbed flow patterns on vascular health and cancer development. Moreover, given that flow shear stress also influences YAP transport and differentiation in stem cells,^[^ ^33^ ^]^ a detailed understanding of mechanistic regulation holds potential in the development of flow‐guided tissue engineering,^[^ ^34^ ^]^ such as tissue‐engineered vascular grafts.^[^ ^35^ ^]^

## 4 Experimental Section

### Cell Culture

Human umbilical vein endothelial cells (HUVECs) were isolated from newborn umbilical cords, as detailly described in the previous study.^[^ ^36^ ^]^ Informed consent was obtained for each donor and all processes were approved by the Beihang University Ethical Committee. The primary HUVECs were cultured in endothelial cell medium (ECM, ScienCell) with 5% FBS, 1% endothelial cell growth supplement (ECGS), and 1% penicillin/streptomycin solution, and incubated at 37 °C under 5% CO_2_. The cells were fed one time a week with a complete change of fresh culture medium and used between 2 to 6 passages.

### Microfluidic Vascular Chip Fabrication

The microfluidic device was fabricated out of polydimethylsiloxane (PDMS, Sylgard 184, Dow Corning) using standard soft lithography (Figure S1a,b, Supporting Information). The microfluidic chip is made up of three layers containing PDMS channel layer, thin PDMS membrane middle layer and glass coverslip supporting layer. To make the PDMS channel layer, PDMS prepolymer with a 10:1 (w/w) mixture of PDMS base and curing agent was casted against a photolithographically prepared master with a designed microchannel made of photoresist (SU8‐2075, MicroChem) on a silicon wafer, and then cured for 2 h in an 80 °C dry oven. The middle layer of thin PDMS membrane was produced by spin‐coating PDMS prepolymer on silanized silicon wafers at 3500 rpm for 50 s on a spin coater and baking on a hot plate at 180 °C for 30 min. The channel layer and thin PDMS membrane were cleaned with dusting compressed air tank, and then treated with oxygen plasma for 50 s to form covalent bonds between them. They were then placed in the oven for 10 min to strengthen the bonding. Finally, the device stripped from silicon was bonded to the glass coverslip in the same manner of oxygen plasma treatment.

Before cell seeding, microfluidic chips were sterilized for 1 h by ultraviolet irradiation in the clean bench, and then the culture channels were coated with 125 µg ml^−1^ fibronectin. Endothelial cells were seeded into the channel and attached to the membrane surface for 1 h at 37 °C under static condition. In all experiments, the density of endothelial cells was controlled at ≈500 cells mm^−2^ to avoid the effects of cell density changes on YAP localization. The attached cells were then filled with ECM by two 3/32″ barbed female Luer adapters (Cole‐Parmer) inserted into the medium injection ports to provide nutrients, which the medium in the Luer adapter was changed every 12 h.

### Control of Flow Shear Stress in Microfluidic System

The control of fluid flow system in the present study consisted of one micro‐syringe pump (Lead Fluid), one electromagnetic pinch valve and a reservoir, which was used in the recent study.^[^ ^36^ ^]^ Medium flow was provided synchronously by the programmable micro‐syringe pumps. The electromagnetic pinch valve was used to switch the fluid flow between injecting into the microfluidic chip and withdrawing from the reservoir. To introduce flow into the microfluidic device, a sterile polypropylene barbed elbow fitting (1/16″, Cole‐Parmer) connected to Teflon tubing was inserted into the inlet port of cell channel. The fluid flow was introduced from the chip's outlet port into the reservoir and later withdrawn to the micro‐syringe pump to create a highly efficient circulation system.

For the unidirectional flow, stable waveforms are used in the control system. For oscillatory flow, the microfluidic chips were connected to the circulation control system input a sinusoidal waveform often used for oscillating flow. The waveform of flow rate was also measured by flow sensor (ElveFlow, Figure S1c, Supporting Information).

### Immunofluorescence and Confocal Microscopy

Cells were fixed in 4% paraformaldehyde (PFA) for 10 min, followed by permeabilization with 0.1% Triton X‐100 in PBS at room temperature and blocking in 5% bovine serum albumin (BSA) for 1 h. Subsequently, cells were treated with primary antibodies at 4 °C overnight and then incubated with appropriate secondary antibodies for 2 h at room temperature. Specifically, YAP was stained with mouse polyclonal anti‐human YAP (sc‐101199, 1:200, Santacruz) and Alexa Fluor 488 goat anti‐mouse IgG H&L (ab150113, 1:200; Abcam). Phalloidin labeled with Alexa Fluor 488‐conjugated phalloidin (YP0052L, 1:200; EVERBRIGHT) was used in conjunction with secondary antibodies. RhoA was stained with rabbit monoclonal anti‐human RhoA (ab187027, 1:200; Abcam) and Alexa Fluor 488 goat anti‐rabbit IgG H&L (ab150077, 1:200; Abcam). mDia1 was stained with rabbit polyclonal anti‐human mDia1 (20624‐1‐AP, 1:200; proteintech) and Alexa Fluor 488 goat anti‐rabbit IgG H&L (ab150077, 1:200; Abcam). Nesprin1 was stained with rabbit monoclonal anti‐human Nesprin1 (ab192234, 1:200; Abcam) and Alexa Fluor 488 goat anti‐rabbit IgG H&L (ab150077, 1:200; Abcam). Cofilin was stained with rabbit monoclonal anti‐human Cofilin (ab283500, 1:200; Abcam) and Alexa Fluor 488 goat anti‐rabbit IgG H&L (ab150077, 1:200; Abcam). Paxillin was stained with rabbit monoclonal anti‐human Paxillin (ab32084, 1:200; Abcam) and Alexa Fluor 488 goat anti‐rabbit IgG H&L (ab150077, 1:200; Abcam). H3K9me3 was stained with Tri‐Methyl‐Histone H3 (Lys9) (D4W1U) Rabbit mAb (13969S, 1:200; Cell Signaling) and Alexa Fluor 488 goat anti‐rabbit IgG H&L (ab150077, 1:200; Abcam). Nuclei were stained with 4′,6‐diamidino‐2‐phenylindole dihydrochloride (DAPI; 1:1000; Sigma–Aldrich) for 10 min. For CM‐DiI staining, the CM‐DiI storage solution (C4060S, 1:1000, Amresco) was added to the microfluidic device and incubated for 5 min at 37 °C in a carbon dioxide incubator. The cells in the microfluidic device were then incubated at 4 °C for 15 min and washed with PBS solution three times. All fluorescence images were z‐stack processed with a step of 0.3 µm, and were acquired using a laser scanning confocal microscopy (SP8X; Leica) with Leica Application Suite software (LAS X version 2.0.0.14332), utilizing a 40× objective lens (NA = 0.6) for general imaging and a 60× objective lens (NA = 1.4) to ensure high spatial resolution when assessing nuclear heights, Paxillin, Nesprin1, and H3K9me3.

### Transfection and Live‐Cell Imaging

15 µg of plasmids for 2 × 10^6^ cells was used, with the transfection mix introduced into a 4 mm electroporation cuvette. The plasmid pcDNA3.1_Lifeact‐mCherry, generously provided by Moritoshi Sato (available from Addgene as plasmid #6730),^[^ ^23^ ^]^ was employed for this experiments. Electroporation was conducted using the Pulse Generator CUY21EDIT II set to a constant voltage of 250 V with a pulse duration of 10 ms. Following electroporation, the cells were plated onto a microfluidic chip and allowed to adhere for 48 hours prior to exposure to a controlled fluid flow. For the imaging process, a Leica SP8X laser scanning confocal microscope equipped with a 40× objective lens was utilized. Lifeact‐mCherry fluorescence was captured using a 561‐nm laser line with appropriate filtering. It was performed z‐stack imaging with a step size of 1 µm over a 12 h period to facilitate XYZ‐t 4D imaging. The actin retrograde flow measurement is based on the open‐source software by Lee et al.^[^ ^37^ ^]^

### Western Blotting

The cells were washed and then lysed in RIPA buffer (Beyotime Biotechnology, China) supplemented with phenylmethyl sulfonyl fluoride (Beyotime Biotechnology, China) and a protease inhibitor cocktail (Beyotime Biotechnology, China). Protein was quantified using the BCA protein assay kit (CwBio, China) according to the manufacturer's instructions. Equal amounts of protein (40 ug) were separated through a 10% SDS‐polyacrylamide gel electrophoresis, and transferred to a PVDF membrane. Membranes were blocked in 5% BSA‐TBST, followed by overnight incubation with appropriate primary antibodies, including pcofilin (1:500 dilution, Cell Signaling Technology, USA), cofilin (1:500 dilution, Cell Signaling Technology, USA), mdia1 (1:800 dilution, ProteinTech, USA), GAPDH (1:1000 dilution, Good Here, China). After membranes were rinsed with TBST buffer, the membranes were incubated with the an HRP‐conjugated secondary antibody at a 1:2000 dilution at room temperature for 2 h. Immunoreactive bands were visualized by chemiluminescence (Applygen, Beijing, China), and the resulting autoradiograms were analyzed by densitometry.

### Latrunculin B Treatment

It was achieved actin caps inhibition in endothelial cells by submerging cells in 30 to 240 nM Latrunculin B (LatB, Abcam) dissolved in DMSO solution (Sigma–Aldrich). To minimize the effects of LatB on conventional fibers rather than the actin cap, the time of LatB treatment was controlled under 1 h. In flow experiments, LatB is added to the ECM medium at the corresponding concentration.

### Brillouin Microscopy Setup

A custom Brillouin microscopy was built to obtain biomechanical images of endothelial cells cultivated in the microfluidic vascular chip based on the recent protocol.^[^ ^38^ ^]^ A 10 mW single longitudinal mode CW 532 nm laser (MSL‐FN‐532, CNI) was utilized as the light source to probe Brillouin scattering. The laser beam was focused into the center of the microfluidic channel by an objective lens (OBJ, NA = 0.6, magnification: 40×), providing a spot size of ≈0.5 µm (transverse) and ≈2 µm (axial). The backward scattered light was collected by the same OBJ, reflected at the polarized beam splitter (PBS), and coupled to the single‐mode fiber (SMF). The microfluidic vascular chip was equipped on a 2D translational stage with 0.5 µm resolution (SC‐200, KOHZU Precision) to perform fast 2D scanning.

The customized Brillouin spectrometer (Figure 1D) has two stages with orthogonally oriented VIPA (Free Spectral Range 30 GHz, LightMachinery). The light coming out of the fiber is coupled in the vertical direction to VIPA 1 through cylindrical lens CL1, and the spectral pattern output from VIPA 1 is projected onto mask 1 (M1) through cylindrical lens CL2. Then, spherical lens L1 couples the pattern on M1 to VIPA 2 in the horizontal direction, and spherical lens L2 projects the spectral pattern at the output of VIPA 2 onto mask 2 (M2). The spectral pattern is imaged by the CMOS camera (iXon, Andor) after relating through a 4f system consisting of spherical lenses and spatial filter, the spectral pattern is imaged by the CMOS camera (ORCA‐Fusion, Hamamatsu). A Matlab software was developed to drive both the stage and the camera simultaneously and output the Brillouin shift data by automatically least square Lorentzian fitting the Brillouin spectra. In each cell nucleus region, it was uniformly selected 10 positions for measurement and calculated the average value to obtain the overall nuclear Brillouin shift (*ν_B_*). To obtain the Young's modulus of the nucleus used in the computational model, it was first calculated the longitudinal modulus M' according to:^[^ ^38^ ^]^

(2) $$M^{\prime }=\frac{\rho_{nuc}\lambda^{2}\nu_{B}^{2}}{4n^{2}}$$

where *ρ_nuc_* refers to the density of the nucleus (1350kg m^−3^), and *λ* is the wavelength of the incident light, n is the refractive index of the nucleus (*n* = 1.38). Then, the longitudinal modulus *M’* to the Young's modulus *E* based on the log‐log liner relationship was transformed:^[^ ^39^ ^]^

(3) $$\log\left(M^{\prime }\right)=a_{B}\;\log\left(E\right)+b_{B}$$

where *a_B_* and *b_B_* are the constant coefficients and equals to 0.081 and 9.37 according to the published study.^[^ ^39^ ^]^

### Preparation and Mechanical Measurements of Gelatin Methacryloyl (GelMA) Hydrogels

At 80°C, different amounts of freeze dried GelMA macromer were dissolved in DPBS containing 0.5% (w/v) 2‐Hydroxy‐4′‐(2‐hydroxyethoxy)−2‐methylpropiophenone (Sigma–Aldrich) as photoinitiator, yielding final GelMA concentrations of 5%, 7.5%, 15%, and 20% (w/v). After pipetting the prepolymer solution into a glass mold, it was subjected to 1000 mw cm^−2^ UV radiation (360–480 nm) for 180 s. In the uniaxial compression test, samples were made as cylinder specimens with diameter of 4 mm and thickness of 8 mm. Brillouin microscopy was then used to assess the stiffness of the samples, which was compared to the Young's modulus derived from compression tests (Figure S4a, Supporting Information).

### Laser Ablation Experiment

This experiment was performed using a femtosecond (fs) pulsed laser ablation system (852 nm, 80 MHz repetition rate, InSight DeepSee). The basal layer of conventional fibers was ablated with 5 repeated cuts at 60 mW laser power. After the ablation, cells were allowed to relax for 30 min before being fixed. Immunofluorescence staining was then used to measure the recoil angle, calculated as the ratio of the short axis to the long axis of the opening ellipse, under different doses of LatB treatments.

### Measurements of Nuclear/Cytoplasmic Ratio of YAP

YR values were measured using image segmentation and quantifying the ratio of YAP intensity inside the nucleus to YAP intensity in cytoplasm. After projecting the mean intensity of the image z‐stack series (50 slices with 0.3 um step) onto a layer in ImageJ 1.5.3q,^[^ ^40^ ^]^ the cytoplasmic YAP from the background using Cellpose was segmented, a pretrained deep learning‐based segmentation method.^[^ ^41^ ^]^ Then, the cell nucleus stained with DAPI via StarDist convolutional neural network was labeled.^[^ ^42^ ^]^ Then, the mean fluorescence intensity of YAP staining in the nucleus and cytoplasm in individual cells were measured respectively. Moreover, during the statistical analysis, the YR values relative to their respective static control groups to eliminate interference from systematic errors was normalized.

### Measurements of Chromatin Compaction with Nuclear DAPI Staining

Here, the microscopy‐based approaches^[^ ^43^ ^]^ to assess the degree of heterogeneity of DNA signal across the nucleus exposed to shear stress by quantifying the coefficient of variation of DAPI intensity was used. After segmenting the nucleus with StarDist convolutional neural network,^[^ ^42^ ^]^ the coefficient of variation (CV) of individual nucleus is calculated as = *σ*/*µ*, where *σ* represents the standard deviation of the DAPI intensity and *µ* represents the mean DAPI intensity.^[^ ^43^, ^44^ ^]^ The segmentation of nucleus with StarDist was achieved in ImageJ 1.5.3q,^[^ ^40^ ^]^ and the standard deviation and the mean value of the DAPI intensity were calculated in MATLAB R2021a.

### Measurements of Total Fluorescence Intensity of Actin Cap

To accurately measure the actin cap's total fluorescence intensity, the procedure by utilizing the StarDist convolutional neural network^[^ ^42^ ^]^ for nuclear segmentation was initiated, applying maximum intensity projection to the DAPI channel. It was then conducted a maximum intensity projection for the phalloidin channel, specifically within the apical planes above the nucleus, to exclude any basal fiber contributions. Then the average F‐actin intensity within each segmented nucleus was calculated. All the above processes were performed with ImageJ 1.5.3q.^[^ ^40^ ^]^

### A 3D Mechanochemical Model for Flow‐Induced YAP Transport

To investigate the mechanochemical interaction among subcellular components under shear stress stimuli, a 3D finite element model (FEM) was constructed that connected fluid flow in the microfluidic channel, mechanical deformation, and biochemical cascade signaling transfer of adhering single cells (see details in Supporting Information Section 1,2). In this study, numerical simulations were carried out using the finite‐element package COMSOL (Burlington, MA), which provides multi‐physics coupling of the laminar flow, the solid mechanics, and the diluted species transport components (Supporting Information, Section 3). The model's extracellular fluid properties were set to match this experimental setup in the microfluidic device. The cellular model was composed of three deformable components: cytoplasm (contains conventional fibers), nucleus, and actin caps, the mechanical properties of which had previously been documented. The biochemical cascade signaling pathways were adapted from the previous numerical models of basal stiffness‐mediated YAP transport.^[^ ^18^ ^]^ The detailed equations and parameters among the model can be found in Tables S1 and S2 (Supporting Information).

### Vertex‐Based Model Simulating Cell Collective Behavior

A vertex‐based model to simulate the collective behavior of endothelial cell monolayers, considering the presence of the actin cap was employed. The vertex model represents the endothelial cell monolayer as a polygonal network, where interconnected polygons correspond to individual cells in contact.^[^ ^45^ ^]^ The computational implementation of the vertex‐based model was performed using CHASTE, a software developed by the University of Oxford, Oxford, UK.^[^ ^46^ ^]^ For a comprehensive understanding of the model, including the detailed equations and parameters utilized, please refer to Supporting Information, Section 4.

### Statistical Analysis

Data was analyzed using GraphPad Prism 9.0 software and were presented as mean ± s.d. Sample numbers and experimental repeats were described in the figure legends. Statistical significance for samples was determined using two‐tailed unpaired Student's t test. Data were considered statistically significant if P < 0.05.

## Conflict of Interest

The authors declare no conflict of interest.

## Author Contributions

T.M. and X.L. conceived, designed, and led the interpretation of the project. T.M. developed the single cell model. T.M. and Y.H. led the development of the collective cell model. H.S., Q.S., C.G., Z.L., D.Z., X.Z., K.L., K.H., J.Z., L.W., M.W., and L.Z. carried out the microfluidic experiments. T.M., F.W., and S.L carried out the optical experiments. S.Y., W.H., X.C., and X.D. provided guidance on experiments. T.M., X.L., P.W., and Y.F. designed the experiments. All authors contributed to the writing of the manuscript.

## Supporting information

Supporting Information

Supplemental Movie 1

Supplemental Movie 2

Supplemental Movie 3

Supplemental Movie 4

Supplemental Movie 5

## Data Availability

The code used to analyze the Brillouin microscopy data is available on the following github link (https://github.com/Leomabuaa/Brillouin‐microscopy). The mechanochemical modeling code is available on the following github link (https://github.com/Leomabuaa/Mechanochemical‐modeling‐of‐endothelial‐cell‐under‐shear‐stress).
