# Supplementary material for: Coupling of Perinuclear Actin Cap and Nuclear Mechanics in Regulating Flow‐Induced Yap Spatiotemporal Nucleocytoplasmic Transport
Source: Adv Sci (Weinh). 2023 Dec 31;11(11):2305867. doi: 10.1002/advs.202305867 (PMC10953556; doi:10.1002/advs.202305867)
Supplement: Supplementary file 1 — Supporting Information [file ADVS-11-2305867-s002.pdf]

ADVANCED SCIENCE
Open Access

Supporting Information

for *Adv. Sci.*, DOI 10.1002/advs.202305867

Coupling of Perinuclear Actin Cap and Nuclear Mechanics in Regulating Flow-Induced Yap Spatiotemporal Nucleocytoplasmic Transport

Tianxiang Ma, Xiao Liu, Haoran Su, Qiusheng Shi, Yuan He, Fan Wu, Chenxing Gao, Kexin Li, Zhuqing Liang, Dongrui Zhang, Xing Zhang, Ke Hu, Shangyu Li, Li Wang, Min Wang, Shuhua Yue, Weili Hong, Xun Chen, Jing Zhang, Lisha Zheng, Xiaoyan Deng, Pu Wang* and Yubo Fan**

Supporting Information

Coupling of Perinuclear Actin Cap and Nuclear Mechanics in Regulating Flow-Induced YAP Spatiotemporal Nucleocytoplasmic Transport

Tianxiang Ma, Xiao Liu[*]*, Haoran Su, Qiusheng Shi, Yuan He, Fan Wu, Chenxing Gao, Kexin Li, Zhuqing Liang, Dongrui Zhang, Xing Zhang, Ke Hu, Shangyu Li, Li Wang, Min Wang, Shuhua Yue, Weili Hong, Xun Chen, Jing Zhang, Lisha Zheng, Xiaoyan Deng, Pu Wang*[*]*, Yubo Fan*[*]

S1. Geometry of the Three-Dimensional Mechanochemical Model

The simulated geometry is one half of the cell and fluid channel, as it is symmetric along the x-z plane shown in Figure S6a. To accurately mimic the real shape of the cell, we conducted CM-Dil staining on the cell membrane and DAPI staining on the cell nucleus (Figure 2c). It should be noted that the geometry of conventional fibers is not constructed directly, but obtained computationally (see section 2.3). Specifically, we utilized Gaussian curves to mathematically fit the long and short axes of the cell membrane (see the schematic of the fitting process is shown in Figure S7a and Figure 2c):

$$Z(x) = A \cdot exp\left(-\frac{x}{c}\right)^2 \tag{S1}$$

Here, *A* represents the peak value of the Gaussian (the heights of cell membrane), which is fitted as 12.03±1.01 μm (n > 30). C represents the standard deviation, and we calculated the boundaries of 95% Gaussian function area to determine the lengths of long axes (110.26±10.12 μm, n > 30) from x-z view and short axes (18.01±1.05 μm, n > 30) from y-z view of the CM-Dil stained cell membrane. Moreover, the dimensions of the nucleus were characterized by its major axis, minor axis, and height, as shown in Figure 2 of the article. Subsequently, the staining data was imported into Creo Parametric 7.0, a powerful computer-aided design (CAD) software, to trace the outlines of the averaged cell membrane and nucleus. Additionally, seven actin cap fibers were constructed within the cytoplasm, and they were connected to the apical surface of the nucleus mimicking the LINC complexes and fixed at the basal surface mimicking the focal adhesions. The fluid channel was established outside the cell, and both the inlet and outlet were extended sufficiently to ensure complete flow development. Furthermore, to avoid any interference with the flow due to boundary effects, the distance between the side walls and the cell membrane was set to be greater than 5 times the short axis of the cell.

S2. Development of the Three-Dimensional Mechanochemical Model

We here present the key features of our three-dimensional cell model for flow shear stress mechanotransduction and YAP transport in section 2. The reactions and parameters of each component are explained in detail in Tables S1-S2.

S2.1 Fluid Flow in the Microfluidic Channel

Our model incorporates a solid cell embedded in the fluid domain, which mimics the cell medium in the microfluidic channel utilized in the experiments (Figure S6a). Specifically, we constructed a rectangular channel filled with homogeneous and incompressible cell medium, which was assumed to exhibit Newtonian rheological properties. The flow of the medium is governed by the three-dimensional incompressible Navier-Stokes equation and the continuity equation:

$$\rho_{fluid}\left(\frac{\partial \boldsymbol{u_{fluid}}}{\partial t} + \boldsymbol{u_{fluid}} \cdot \nabla \boldsymbol{u_{fluid}}\right) = \mu \nabla^2 \boldsymbol{u_{fluid}} - \nabla p \qquad \text{(S2)}$$

$$\nabla \cdot \boldsymbol{u_{fluid}} = 0 \qquad \text{(S3)}$$

where $\mathbf{u_{fluid}}$ represents the fluid velocity and p represents the pressure. The fluid density (ρ_{fluid}) and viscosity (μ) are set to 1050kg m^{-3} and 0.95×10^{-3}Pa·s, respectively, to maintain consistency with the experimental conditions. The channel wall and cell membrane surface were treated as no-slip boundaries. Additionally, we adjusted the inlet flow velocity boundary to ensure that the flow shear stress at the channel wall aligns with the experimental conditions, while the flow shear stress on the cell membrane surface will exceed this value (Figure S6b). Furthermore, the outlet pressure boundary was set to be a zero pressure, which also aligns with the experimental conditions.

S2.2 Fluid-Structure Interaction at Cell Membrane Surface

We integrated fluid flow with cellular solid mechanics by performing fluid-structure interaction (FSI) analysis to accurately capture the transmission of flow shear stress. The FSI coupling is applied at the cell membrane surface (as shown in the FSI interface in Figure S6a), which accounts for both fluid forces acting on the solid cell and the transmission of cell deformation-induced velocities to the fluid. The following equations describe the interactions between the fluid and solid domains at FSI interface:

$$\boldsymbol{\Gamma} = -\mathbf{pI} + \mu(\nabla \mathbf{u_{fluid}} + (\nabla \mathbf{u_{fluid}})^{\boldsymbol{T}}) \qquad \text{(S4)}$$

$$\boldsymbol{\Gamma} \cdot \boldsymbol{n} = \boldsymbol{\sigma} \cdot \boldsymbol{n} \qquad \text{(S5)}$$

$$\boldsymbol{v_{wall}} = \frac{\partial \mathbf{u_{solid}}}{\partial t} \qquad \text{(S6)}$$

Equation (S4) describes the transfer of stress from the fluid to the solid domain. Here, $\boldsymbol{\Gamma}$ is the stress tenor in the fluid domain, and $\mathbf{I}$ refers to the identity matrix. Equation (S5) represents

the normal stress balance condition on the FSI interface, where n is the unit normal vector, and **σ** is the stress tensor in the solid domain. In addition, in Equation (S6), $\mathbf{v}_{\mathbf{wall}}$ represents the velocity of the FSI interface, which acts as a moving wall for the fluid, while $\mathbf{u}_{\mathbf{solid}}$ represents the displacement of the solid cell. By solving these equations using the arbitrary Lagrangian-Eulerian (ALE) method, which combines the Eulerian description of fluid flow with the Lagrangian description of solid mechanics, the flow shear stress transmission can be effectively simulated.

S2.3 Solid Mechanics of Deformable Cell with Subcellular Components

In our three-dimensional model, the solid domain comprises the cytoplasm (contains conventional fibers), nucleus, and actin cap. Within this domain, both the cytoplasm and nucleus undergo only passive deformation in response to external forces, while the conventional fibers and actin cap are capable of experiencing both passive cytoskeletal stiffness and myosin-driven contractile stress.[1] Firstly, we treated the cytoplasm as an isotropic linear elastic material with a constitutive relationship described by the Hooke's law:

$$\boldsymbol{\sigma} = \frac{E_{cyto}}{1+\nu}\boldsymbol{\varepsilon} + \frac{\nu E_{cyto}}{(1+\nu)(1-2\nu)} tr(\boldsymbol{\varepsilon})\boldsymbol{I} \tag{S7}$$

Here, E_{cyto} represents the Young's modulus of the cytoplasm, ν represents the Poisson's ratio. The values for all parameters in our model were summarized in Table S2. The first term of Equation (S7) represents the linear elastic response of the cytoplasm to the strain tensor **ε**, while the second term represents the volumetric response.

Notably, the cytoplasm domain contains conventional fibers. In this paper, the orientation of conventional fibers is established by assessing the concentration gradient of a dimensionless scalar U, which diffuses from the basal surface of the cytoplasm towards the nuclear membrane and governed by the diffusion equation. This method is similar to the approach employed in the works of Novev et al.[2] and Bakir et al.,[3] which is mathematically described as:

$$\xi = \frac{-\nabla U}{|-\nabla U|} \tag{S8}$$

Here, ξ represents the vector field of conventional fibers as shown in the left panel of Figure S6c. Similarly, the vector field of the actin cap is determined using Equation (S8), where the dimensionless scalar U diffuses from one focal adhesion to the other within the actin cap domain (right panel of Figure S6c). The cytoskeletal stress is assumed to exist only in the direction of the fibers, which includes contributions from F-actin-driven passive stiffness (K_F), myosin-driven contractile stress (σ_M), and the volume fraction of fibers in the unit volume of the domain (ρ),[1] which can be calculated as:

$$\sigma = \rho\sigma_i = \rho(k_F\varepsilon + \sigma_M) \quad \text{(S9)}$$

To mimics the dynamic formation of conventional fibers, ρ is assumed to be the first-order functions of the F-actin (further details are provided in the subsequent section of this Supporting Information Text). Regarding the actin cap, ρ is obtained from experimental measurements using the Hill equation fitting,[4] which is governed by:

$$H = H_0\left(1 + \frac{\alpha t^4}{t^4+\beta^4}\right) \quad \text{(S10)}$$

Here, H_0, α, and β are parameters that determine the shape of the Hill equation, and t represents the flow time. The specific values for each flow condition can be found in Figure S7b. During the fitting process, special care was taken to ensure that the R-square value for each group fit exceeded 0.95, ensuring the accuracy of the fitting procedure. It is important to note that the decision to utilize experimental data for fitting the actin cap formation equation is due to the complex signaling pathways involved in this process. For instance, the actin cap formation in response to flow involves intricate interactions with various proteins, such as zyxin-mediated formation at low shear stress and talin-mediated formation at high shear stress.[5] These complexities make it challenging to describe the actin cap formation with explicit mathematical expressions in the current model.

Regarding the cell nucleus, we considered it as an isotropic viscoelastic material, and its constitutive relationship is described by the Maxwell model as shown in Equation (S11):

$$\dot{\varepsilon} = \frac{\dot{\sigma}}{E_{nuc}} + \frac{\sigma}{\tau_r} \quad \text{(S11)}$$

Here, E_{nuc} represents the Young's modulus of the nucleus, and τ_r represents the relaxation time of the nucleus (The values for all parameters in our model were summarized in Table S2).[6]

As the boundary conditions for the solid domain, the bottoms of the compartments were fixed in all directions, including the cell basal surface and focal adhesion of actin caps, replicating the adhesion of the cell to the substrate. The surfaces of nucleus and actin cap are in contact with each other. The contact conditions were assumed as tied-type contact methods to mimics the present of LINC.

S2.4 Transport Model of YAP-Mediated Mechanotransduction

To comprehensively understand and predict the nucleocytoplasmic shuttling of YAP, our study integrates both the involved biochemical pathways and mechanical regulations. Regarding the biochemical regulation, we consider essential components associated with YAP mechanosensing, namely RhoA, rho-associated kinases (ROCK), formin mDia1 (mDia), LIM kinase (LIMK), and Cofilin pathways, as depicted in Figure 2b in the manuscript. The

transport processes of the activating and deactivating components of these pathways are governed by reaction-diffusion equations derived from previously validated model.[4]

For the upstream component RhoA in our model, we account for its transport within the cytoplasm, its activation through FAK phosphorylation near focal adhesions, and its activation in response to flow shear stress at the apical plasma membrane. The governing equations describing these processes are as follows:

$$\frac{\partial C_{RhoA}}{\partial t} = D_{RhoA}\Delta C_{RhoA} + n_r k_{fk\rho}\left(\gamma C_{pFAK}{}^{5} + 1\right)C_{Rho} - k_{d\rho}C_{RhoA} \quad \text{(S12)}$$

$$-D_{RhoA}(\boldsymbol{n} \cdot \nabla C_{RhoA})|_{PM-apical} = T \cdot R_{a\rho} \quad \text{(S13)}$$

Here, C_{RhoA}, D_{RhoA} represents the concentration and diffusion coefficient of RhoA, respectively. The second term in Equation (S12) describes the activation of RhoA due to FAK phosphorylation (pFAK) near focal adhesions. Here, n_r, $k_{fk\rho}$, γ, and pFAK concentration (C_{pFAK}) collectively determine the activation rate of RhoA due to pFAK, and $k_{d\rho}$ represents the deactivation rate of RhoA.[4a, 7] In addition, in Equation (S13), T represents the thickness of plasma membrane,[8] and $R_{a\rho}$ represents the RhoA activation at plasma membrane with kinetics expressed by Hill equation:[9]

$$R_{a\rho} = R_{max,\rho}\frac{\tau}{\tau + a}C_{Rho} \quad \text{(S14)}$$

Where $R_{max,\rho}$ represents the maximum activation rate, τ denotes shear stress magnitude, C_{Rho} is Rho concentration (deactivation form of RhoA), and a is shear stress value when the activation rate of RhoA reaches $R_{max,\rho}/2$. FAK is known to be activated and converted to pFAK by the stress at focal adhesions, whose transport and activation are modeled as:

$$\frac{\partial C_{pFAK}}{\partial t} = D_{pFAK}\Delta C_{pFAK} + k_f C_{FAK} - k_{df}C_{pFAK} \quad \text{(S15)}$$

$$-D_{pFAK}\left(\boldsymbol{n} \cdot \nabla C_{pFAK}\right)\Big|_{PM-basal} = T \cdot R_{pFAK} \quad \text{(S16)}$$

In Equation (S15), C_{pFAK} and D_{pFAK} represent the concentration and diffusion coefficient of phosphorylated FAK, respectively. k_f and k_{df} model the baseline phosphorylation rate and deactivation rate of FAK, respectively. Notably, Equation (S16) describes the activation rate of pFAK in response to cytoskeletal stress, and the stress-sensitive activating rate R_{pFAK} is modeled as a second-order Hill equation:[4b]

$$R_{pFAK} = R_{max,pFAK}\frac{\sigma^2}{\sigma^2 + C^2}C_{FAK} \quad \text{(S17)}$$

Here, $R_{max,pFAK}$ is the maximum activation rate due to stress (σ) in FA, C is the value of stress when the activation rate of FAK is $R_{max,pFAK}/2$. This expression is analogous to the previously published mathematical description of cytoskeletal stress equivalence based on substrate stiffness.[4a] Additionally, to account for the more rapid FAK phosphorylation at focal

adhesions within the actin cap,[10] we assumed a tenfold amplification of R_{pFAK} at actin cap associated focal adhesions.

Based on the activation of RhoA, we simulated the associated actin fibers reorganization process using reaction-diffusion equations. This process involves the mDia-actin pathway, the ROCK-LIMK-cofilin-actin pathway, and the ROCK-myosin pathway. The mathematical formulations and parameter choices for these biochemical conversions were derived from the studies of Scott et al.[4a] and Sun et al.,[4b] and are comprehensively explained in Table S1-S2. Specifically, the formation (polymerization) of F-actin is determined by the concentrations of mDia and cofilin, as described by the following equation:

$$\frac{\partial C_{F-actin}}{\partial t} = D_{F-actin}\Delta C_{F-actin} + k_{ra}\left(\lambda C_{mDia_A} + 1\right)C_{G-actin} - \left(k_{dep} + k_{fc1}C_{Cofilin}\right)C_{F-actin} \qquad \text{(S18)}$$

Here, $C_{F\text{-}actin}$ and $D_{F\text{-}actin}$ represent the concentration and diffusion coefficient of F-actin, respectively. The second term describes the polymerization rate of F-actin, driven by the interaction between G-actin and activated mDia. The third term represents the depolymerization rate of F-actin due to cofilin. Next, we employ the concentration of F-actin divided by its maximum concentration under unidirectional flow as the volume fraction (ρ) in Equation (S9). This design allows us to simulate the mechanical effects resulting from F-actin polymerization.

In addition to the passive stiffness determined by F-actin, we further considered the active contractile stress contributed by activated myosin (see σ_M in Equation (S9)), and σ_M is simulated as a linear function of the concentration of activated myosin (C_{MyoA}), i.e., $\sigma_M = k_{act}C_{MyoA}$. The myosin activation is described by the following equation:

$$\frac{\partial C_{MyoA}}{\partial t} = D_{MyoA}\Delta C_{MyoA} + k_{mr}\left(\kappa C_{ROCK_A} + 1\right)C_{Myo} - k_{dmy}C_{MyoA} \qquad \text{(S19)}$$

In Equation (S19), D_{MyoA} represents the diffusion coefficient of activated myosin. The second term in the equation describes the effects of ROCK activation on myosin activation, while the third term represents the deactivation rate of activated myosin. The intricate interplays among the inactivated/activated mDia, ROCK, LIMK, Cofilin, myosin, and actin is detailed in Table S1-S2.

Finally, we have incorporated a comprehensive set of reactions to model the transport of YAP. This model not only considers the YAP dephosphorylation induced by F-actin and activated myosin[11] but also accounts for the mechanosensitive regulation resulting from the cytoskeletal stress acting on the nucleus.[12] Specifically, the rate of YAP dephosphorylation is modeled as a product of F-actin and myosin concentrations,[4a] while YAP's nuclear localization or cytoplasmic retention is governed by the export and import rates of

dephosphorylated YAP.[4a, 12] The kinetics of YAP shuttling between the nucleus and the cytoplasm can be described by the following equations:

$$\frac{\partial C_{YAP}}{\partial t} = D_{YAP}\Delta C_{YAP} + \left(k_{CN} + k_{CY}C_{F-actin}C_{MyoA}\right)C_{pYAP} - k_{NC}C_{YAP} - J_{YAP,NM} \quad \text{(S20)}$$

Here, C_{YAP} and D_{YAP} represent the concentration and diffusion coefficient of YAP (representing the dephosphorylated YAP), respectively. The second term considers the dephosphorylation process of phosphorylated YAP (pYAP). The last term describes the nucleocytoplasmic shuttling of YAP. In this paper, we modeled it as a function of nuclear membrane equivalent strain, reflecting nuclear pore stretch:

$$J_{YAP,NM} = k_{in}\frac{\varepsilon_{NM}}{\varepsilon_{NM}+\varepsilon_{NM,T}}C_{YAP} - k_{out}C_{YAP_{nuc}} \quad \text{(S21)}$$

where k_{in} and k_{out} is the first-order constant modulating the rate of YAP nuclear import and export. C_{YAP} and C_{YAPnuc} are the concentration of cytoplasmic and nuclear YAP, and ε_{NM} represents the nuclear membrane equivalent strain, which is calculated as $\varepsilon_{NM} = [2/3\ (\varepsilon_{xx}^2 + \varepsilon_{yy}^2 + \varepsilon_{zz}^2 + 2\varepsilon_{xy}^2 + 2\varepsilon_{xz}^2 + 2\varepsilon_{yz}^2)]^{1/2}$. $\varepsilon_{NM,T}$ is the value of strain when the import rate reaches its half maximum. The deformation of nuclear membrane is determined by the stress transmitted from actin fibers shown in Equation (S9) and the nuclear stiffness. The relationships between nuclear stiffness, flow shear stress and time are fitted from the Young's modulus calculated from the measured Brillouin shift with Hill equation (Figure S7c), which is similar to the method utilized for fitting actin cap volume fraction.

Equation S21's form is similar to the previously published model that used substrate stiffness, nuclear pore complex, and Lamin concentration to equivalently represent nuclear deformation. In this equation,[4a] the first term reflects the promotion of YAP nuclear entry by nuclear pore stretch, while the second term sets the YAP export rate as a first-order rate, demonstrating its relatively weak mechanical sensitivity.[2, 12a] The detailed mechanisms and parameter values for this nucleocytoplasmic shuttling process are elucidated in Tables S1-S2. The sensitivity analysis of key parameters is provided in Figure S9b.

S3. Simulation Methods of the Three-Dimensional Mechanochemical Model

The mechanochemical model was implemented using the finite element package COMSOL (Burlington, MA), a widely recognized software that provides robust capabilities for multi-physics simulations. We leveraged COMSOL's ability to couple laminar flow, solid mechanics, and diluted species transport components, thereby capturing the comprehensive physics involved, as indicated in Table S1. For the implementation, we employed the fully

coupled approach and utilized the MUMPS Direct solver with relative and absolute error tolerances set at 1×10^{-3} and 5×10^{-5}, respectively.

To simulate the unidirectional flow, we conducted simulations for 24 or 72 hours, using time steps of 0.1 seconds that grew exponentially to accelerate calculations. For oscillatory flow simulations, we shortened the time to 24 hours to capture the process of actin cap formation and nucleus stiffness alteration. The calculations were performed using uniform 0.25-second time steps for a total of 8.64 seconds to expedite the process. All simulations were run for a total of 100 seconds to obtain a steady state. In addition, the initial conditions of all transport components are kept constant with the published chemical model.[4a]

To mesh the entire geometric model, we employed tetrahedral meshing with a total of 133,364 elements (Figure S6a). To ensure the alignment and non-overlapping nature of grid nodes, various subdomains with different spatial sizes and transport properties shared topology. In the boundary layer, we set the element adjacent to the endothelial membrane surface to be 0.15 μm, gradually growing over 5 layers. We performed mesh independence tests, using meshes of increasing fineness until no differences were observed between meshes of two consecutive refinement steps. This ensured that our simulation results were not affected by the mesh density.

To enhance reproducibility and accessibility, the COMSOL modeling file is publicly available on Github (see Data Availability).

S4. Vertex-Based Model Simulating Collective Cell Behavior

We employed the vertex model to represent the endothelial cell monolayer as a polygonal network, where interconnected polygons represent individual cells in contact.[13] The vertex dynamics follow a mechanical force balance, with each vertex position satisfying the overdamped dynamics equation:

$$\gamma \frac{dr}{dt} = F_i^{passive} + F_i^{active} \tag{S22}$$

where γ denotes the friction coefficient between cells and the substrate. The passive force is described as $F_i^{passive} = -\partial U_f / \partial r_i$, and the energy function U_f is defined as:

$$U_f = \frac{1}{2} \sum_J \left[K_a \left(A - A_{0J} \right)^2 + K_c {L_J}^2 \right] + \sum_{\langle i|j \rangle} \Lambda l_{ij} \tag{S23}$$

where A, L_J, and l_{ij} represent cell area, perimeter, and bond length, respectively. r_i represents the position of vertex i. In addition, A_{0J} refers to the preferred cell area, K_a and K_c refer to the area and perimeter stiffness, and Λ represents the bond tension. Consequently, the three terms on the right-hand side correspond to area elasticity stress, perimeter elasticity stress, and

intercellular tension, respectively. The active force $F_i^{active}=F\,p_i$ accounts for the traction forces of magnitude F exerted by the cells abutting at vertex i and in the average direction of their polarities $p_i = \sum_{\langle\alpha|i\rangle} p_\alpha/M_\alpha$. The cell polarity p_α directs the traction force applied to cell α by the surrounding matrix. The time evolution of each cell polarity obeys the random rotation dynamics $dp_\alpha/dt=\phi(t)$,[14] with $\phi(t)$ representing the white Gaussian noise.

For laser ablation experiment simulation, we eliminated four central cells in the middle to simulate the laser cutting. For flow shear stress simulation, we added a bulk stress in the vertex model to reflect the effect of shear stress on cell morphology based on the framework of Tlili,[15] which is assessed through the work of Lin et al.[16] Aligning with the flow direction, the dimensionless bulk stress $\tilde{\sigma}_{ACs}$ is transformed into the force applied to each vertex as:

$$F_i^{AC}(\tilde{\sigma}_{\mathrm{ACs}}) = -R_i \cdot \tilde{\sigma}_{\mathrm{ACs}} \tag{S24}$$

$$R_i = \frac{1}{2}(r_{i+1} - r_{i-1}) \times \hat{z} \tag{S25}$$

where R_i is the normal vector of the line $l_{(i+1,i-1)}$ connecting vertex i+1 and i-1. r_i+1 and r_i-1 represent the position of vertex i+1 and i-1, and $\hat{z}$ represents the unit vector perpendicular to cell monolayer. The topological defects in the orientation field are detected by following the presented method.[17] In addition, the nuclear strain is calculated based on our proposed minimal model using the stress within actin cap and conventional fiber. The stress within conventional fiber is assumed to be the summation of cellular elasticity stress and intercellular tension. The computational method for the vertex-based model was implemented in CHASTE (University of Oxford, Oxford, UK).[18] We used the following dimensionless values in the simulation to match the behavior of endothelial cell alignment: A_{0J}=1, K_a=1, K_c=0.2, p_0=-Λ/K_c=3.6, $\tilde{\sigma}_{ACs}$=0.6, F=0.05, γ=1.

S5. Regulation of cell monolayer behavior by actin cap formation

In cell monolayers, topological defects of cell alignments have been reported to influence spatial varying YAP activity, mediating cell death and extrusion processes.[19] Considering the close relationship between actin stress fiber and cell alignments under flow, and the impact of actin reorganization on collective cellular mechanotransduction,[20] we integrated our single-cell mechanochemical model with the vertex model to investigate the regulatory role of actin cap in cell monolayer behavior with focus on nucleus membrane strain, which is the key factor for YAP dynamics. To achieve this, we modified the vertex model to consider the flow-induced formation of actin cap. The cell shapes and stress in conventional fibers (σ_{CFs}) are

determined by their packing topology.[21] The stress in actin cap (σ_{ACs}) is implemented by an anisotropic bulk stress within each cell (Figure S11). We found that upon actin cap forming, the vertex model successfully reproduced the experimentally measured endothelial cell aspect ratio and alignments under flow (Figure S11b). To achieve a relatively general and concise model of various mechanical stimulations such as flow shear, substrate stretching and stiffness, we derived a minimal model of nuclear membrane strain based on our 3D modeling and integrated it with the vertex model (Figure S11a), which is expressed as:

$$\varepsilon_{NM} = \frac{\sigma_{CFs}+\rho\sigma_{ACs}}{E_{nuc}+\rho E_{ACs}} \tag{S26}$$

Here, E_{nuc} represents nuclear stiffness, ρ denotes actin cap formation, and E_{ACs} corresponds to actin cap stiffness. The apparent stiffness, $E_{nuc}+\rho E_{ACs}$, is determined by both the nucleus and the actin cap. To validate the reliability of Equation (S26), we applied the 3D model's simulated time sequences of stress and successfully reproduced the initial rise and decline in nuclear strain (Figure S11c). In addition, the Equation (S26) captures the relationship between nuclear stiffness, conventional fibers stress, and nuclear membrane strain when keeping ρ as constant (Figure S11d), consistent with our 3D model's results and previous findings.[2, 4a, 12] The influence of actin cap on nuclear membrane strain depends on the ratio between its active stress and stiffness (Figure S11e). A larger σ_{ACs} to E_{ACs} ratio leads to increased nuclear strain as actin cap form, potentially corresponding to actin cap formation under short-term mechanical stimuli, as observed in periodontal ligament cells responding to flow within 1 hour[22] and cells exposed to substrate stretching and stiffness,[23] leading to YAP nuclear import. In contrast, a smaller ratio decreases nuclear strain, potentially reflecting long-term actin cap formation, such as in endothelial cells responding to unidirectional flow and cells experiencing prolonged stretching,[24] leading to YAP nuclear export.

The vertex model results show that the inhibition of actin cap in cell monolayer results in an increased nuclear membrane strain (Figure S11f), which agrees with our experimental results of actin cap inhibition promotes YAP nuclear import (Figure 1i). Then, we selectively inhibited actin cap formation in individual cells and found that the localized actin cap irregularities affect collective cells alignments (Figure S11h). Notably, the simulation results predicted that the localized actin cap irregularities can induce topological defects and spatially heterogeneous nucleus membrane strain in cell monolayer (Figure S11g). The defects probability and coefficient of variation for spatial distribution of nucleus membrane strain exhibited a significant increase when the target cell underwent complete actin cap inhibition. This increase progressively diminished as the degree of actin cap inhibition decreased (Figure S11i and j). Collectively, our vertex model analysis predicts the potential role of actin cap in

modulating collective cell alignments and spatial distribution of nucleus membrane strain within the cell monolayer.

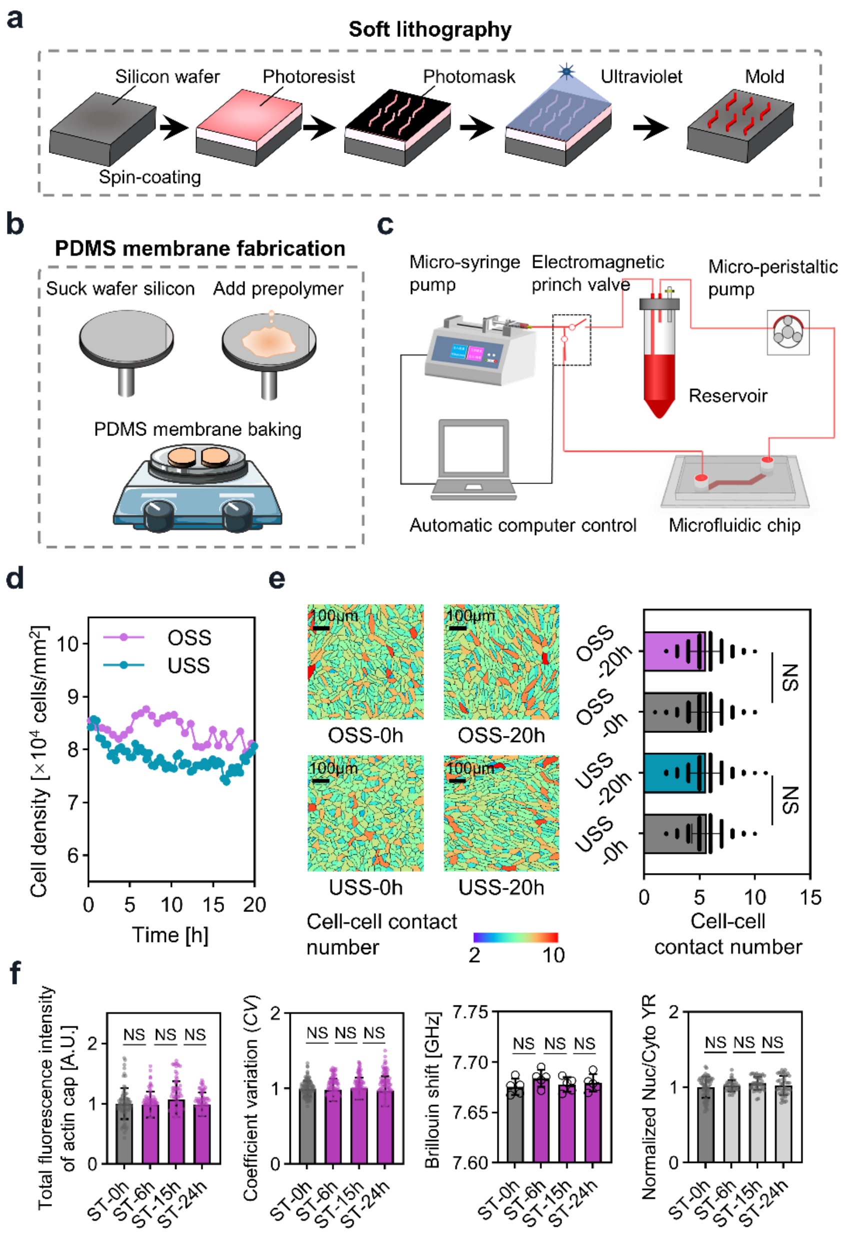

Figure S1. Microfluidic chip fabrication, computer-controlled automatic perfusion system, and monitoring of cell-cell contact under flow application. (a-b) Schematic representation of the soft lithography process and PDMS membrane fabrication. (c) Schematic representation of the computer-regulated automatic perfusion system. (d) Under flow conditions, cell density consistently fluctuates between 700-900 cells/mm^2 (unidirectional flow shear stress, USS: 12 dyne cm^{-2}; Oscillatory flow shear stress, OSS: 0±12 dyne cm^{-2}, 1Hz). (e) Both USS and OSS exhibit consistent cell-cell contact numbers (3 independent experiments, n > 80 cells). (f) Total fluorescence intensity of actin cap, coefficient of variation, Brillouin shift, and YAP nuclear-cytoplasmic ratio (YR) of control cells cultured for 24 hours under no mechanical stimulation. NS, not significant.

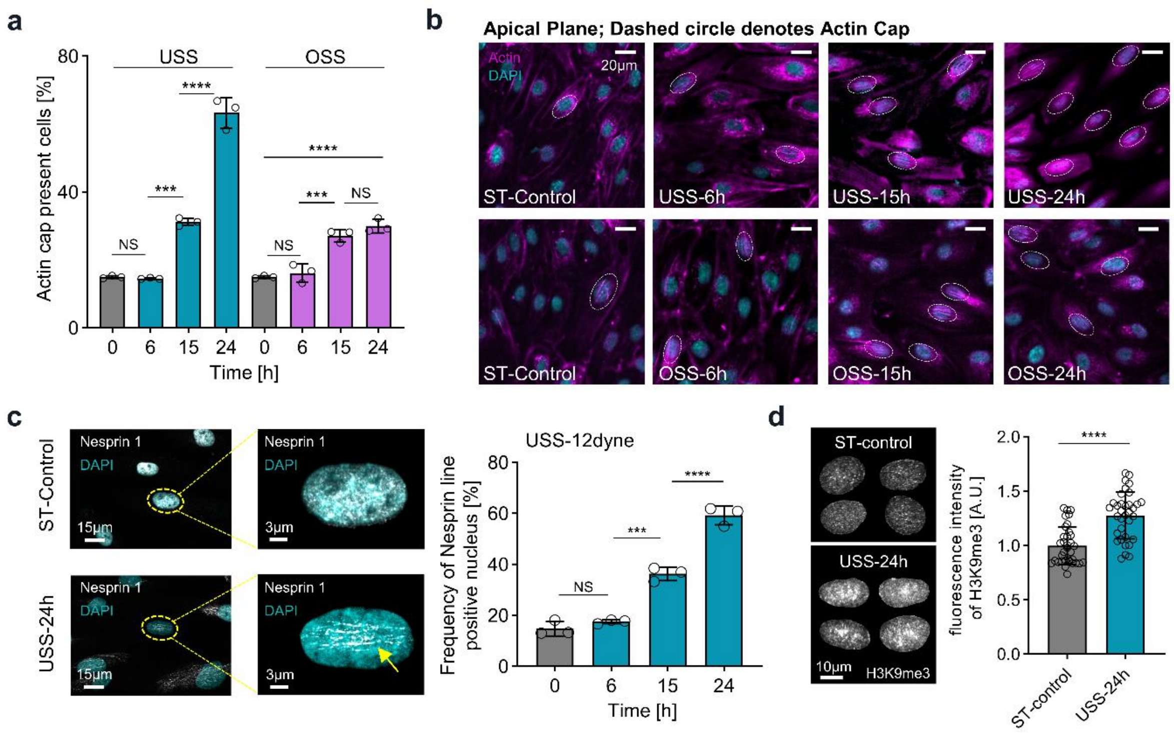

Figure S2. Complementary results of actin cap formation and nuclear stiffening under flow shear stress. (a) Flow-dependent formation of actin cap under USS of 12 dyne cm^{-2} and OSS of 0±12 dyne cm^{-2}, 1Hz (3 independent experiments n > 80 cells). (b) Apical view of actin cap formation under USS of 12 dyne cm^{-2} and OSS of 0 ± 12 dyne cm^{-2}, 1Hz. The actin cap present cells are marked with dashed circles. (c) Nuclear Nesprin lines in static control and USS of 12 dyne cm^{-2}. Yellow arrows indicate the presence of the Nesprin line in the nucleus. (d) Enhanced fluorescence intensity of H3K9me3 within the nucleus in response to USS of 12 dyne cm^{-2} (normalized by the statics control). The data is derived from 3 independent experiments, with more than 30 cells analyzed (n > 30). All data are shown as mean ± s.d. ***$p < 0.001$; ****$p < 0.0001$; NS, not significant.

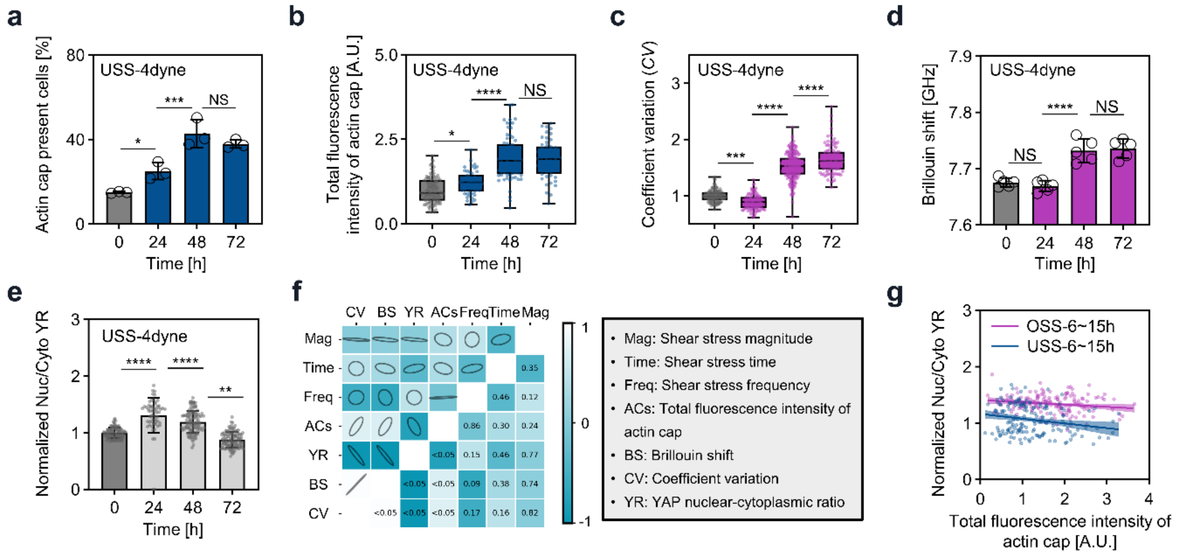

Figure S3. The dynamics of actin cap formation, nuclear stiffness, and YAP localization under flow shear stress. (a-e) Time-dependent variations in actin cap present cells, total fluorescence intensity, coefficient of variation, Brillouin shift, and YR under USS of 4 dyne cm^{-2} (3 independent experiments n > 80 cells, 5 independent experiments in Brillouin tests). (f) Correlation matrix analysis among shear stress magnitude, time, frequency, total fluorescence intensity, coefficient variation, and Brillouin shift. The upper half of the matrix displays correlation, while the lower half displays significance. (g) Linear regression of YR with respect to total fluorescence intensity of actin cap under 6 to 15 hours USS of 12 dyne cm^{-2} ($p<0.01$) and OSS of 0 ± 12 dyne cm^{-2}, 1Hz ($p<0.01$). All data are shown as mean ± s.d. *$p<0.05$; **$p<0.01$; ***$p<0.001$; ****$p<0.0001$; NS, not significant.

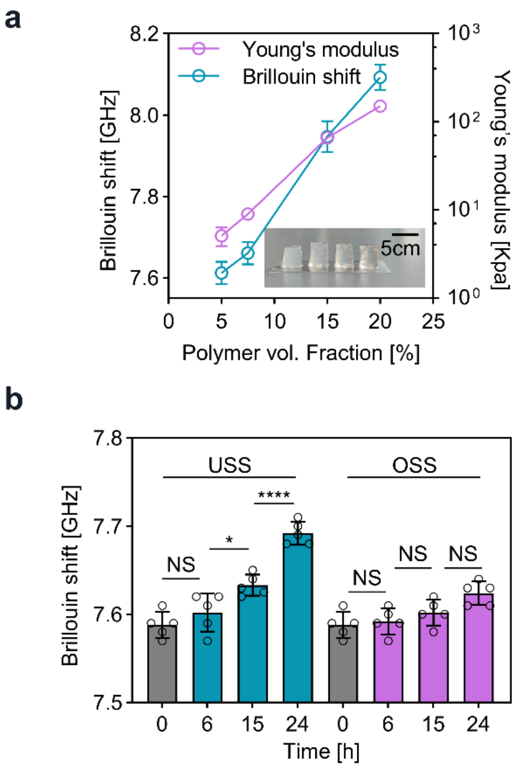

Figure S4. Supplemental results of Brillouin measurements. (a) Comparison between the Brillouin shift observed using Brillouin microscopy and the Young's modulus obtained from uniaxial compression tests. Both metrics exhibit a positive correlation with the GelMA fraction. (b) Under unidirectional flow (USS, 12 dyne cm^{-2}), there is a significant increase in cytoplasmic stiffness concurrent with nuclear stiffening and actin cap formation. When subjected to oscillatory flow (OSS, 0±12 dyne cm^{-2}, 1Hz), there is only a slight increase in cytoplasmic stiffness and not statistically significant (n=5 for each condition). All data are shown as mean ± s.d. *$p<0.05$; ****$p<0.0001$; NS, not significant.

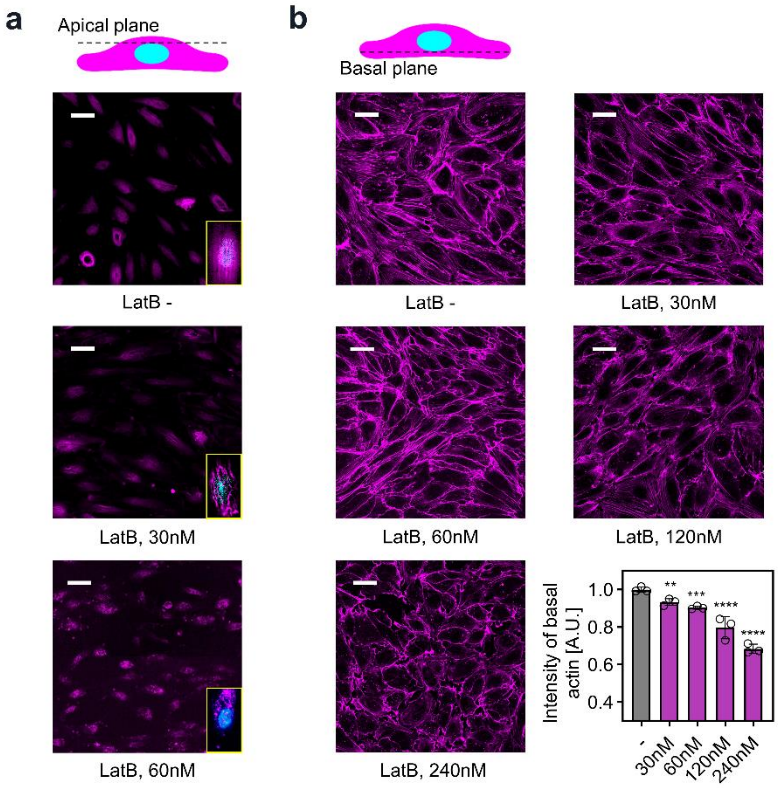

Figure S5. The effects of latrunculin B treatment on the actin cap and basal conventional fiber. (a) Effects of latrunculin B in various doses ranging from 30 to 60nM on actin cap. (b) Effects of latrunculin B in various doses ranging from 30 to 240nM on basal conventional fibers, and the quantification of the total intensity of basal actin fibers (normalized to none LatB treatment control, n=3). Scale bars, 40μm. Data are shown as mean ± s.d. **p<0.01; ***p＜0.001; ****p＜0.0001.

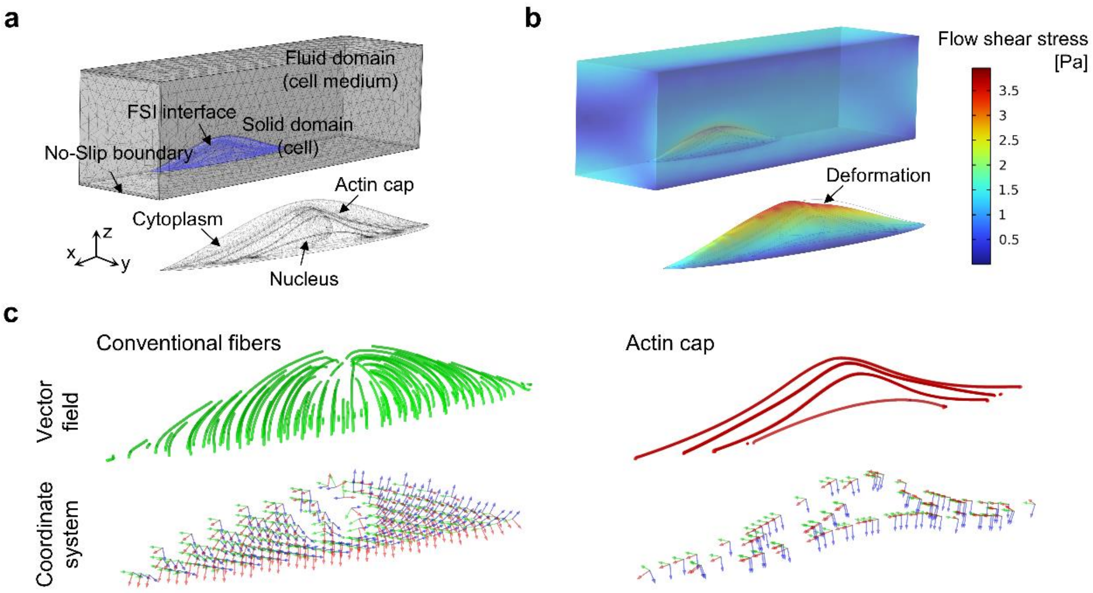

Figure S6. The mechanical details of the extracellular and intracellular environment. (a) The calculating domains are divided to the fluid domain (corresponds to the medium within the channel), solid domain (cytoplasm, nucleus, and actin cap). The fluid domain and solid domain are fully coupled to achieve fluid-structure analysis. (b) The flow shear stress in the channel and the deformed cellular plasma membrane under the condition of unidirectional flow shear stress with 12 dyne cm^{-2}. (c) The anisotropic direction of conventional fibers, which is calculated through the diffusion potential of F-actin, and the anisotropic direction of actin cap derived from experimental observation.

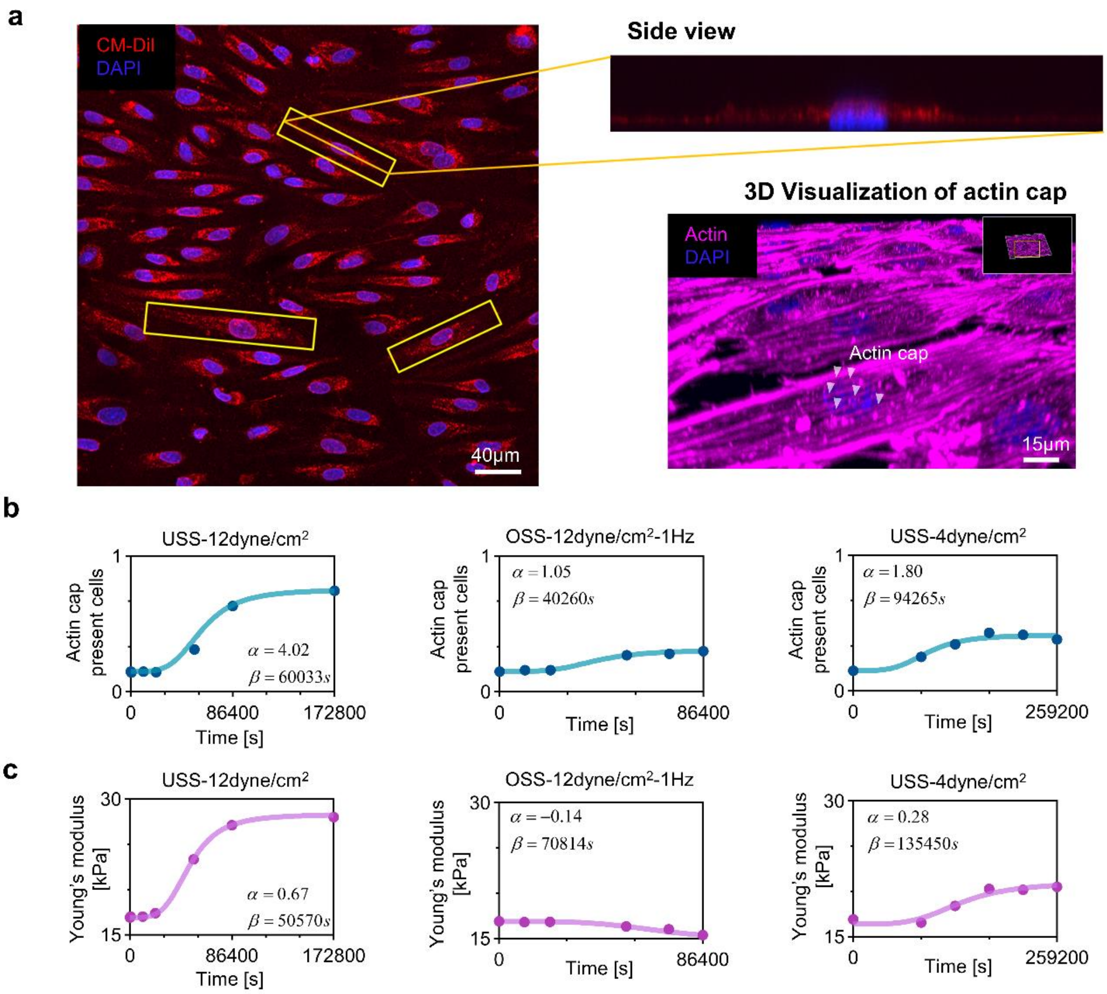

Figure S7. Illustrates the experimental data-driven fitting of cell geometry, actin cap, and nuclear stiffness. (a) The workflow used to obtain cell membrane shape through CM-Dil staining and the side view used for Gaussian curve fitting. Three-dimensional visualization of actin cap structure (actin caps are indicated by white arrowheads, unidirectional flow shear stress, 12 dyne/cm^2, 24 hours). (b) The relationships between actin cap present cells, flow time and shear stress condition (R-square value of each group fit exceeds 0.95), which is utilized as the volume fraction (ρ) for actin cap formation in the model. (c) The relationships between nuclear stiffness, flow time and shear stress condition (R-square value of each group fit exceeds 0.95), which is utilized as the nuclear stiffness (E_{nuc}) in the model. Specifically, α and β describe the shape of the Hill equation, which are shown in the corresponding subgraphs.

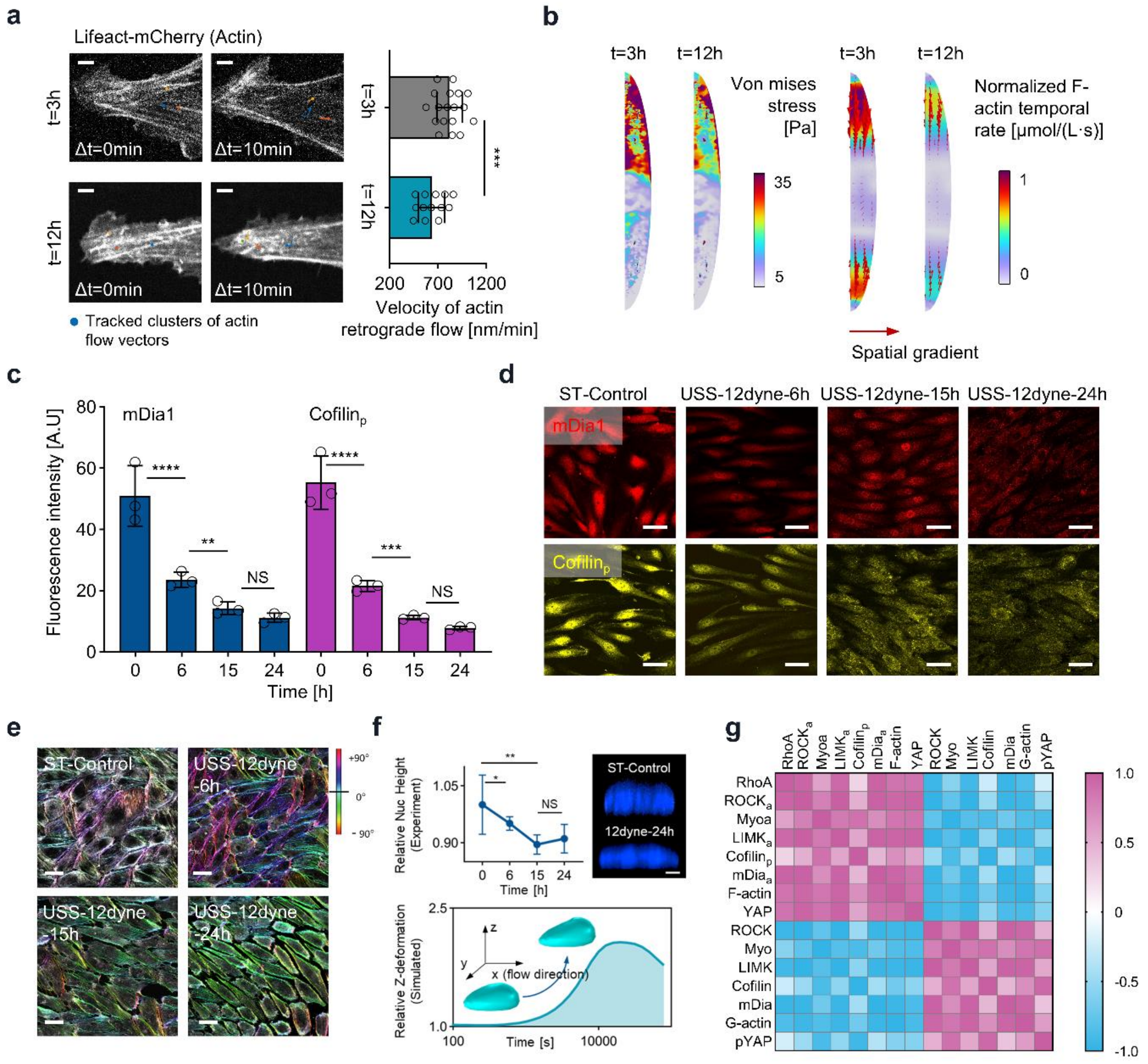

Figure S8. Validation of signaling pathways involved in the model. (a) Lifeact imaging of actin at 3-hour flow and 12-hour flow (left, the trace of marked points represents the movement of actin flow vectors cluster between 10-min interval), the decrease in the mean velocity of actin retrograde flow (n > 12, 3 independent experiments). (b) Simulated Von mises stress at 3-hour and 12-hour flow (left), and F-actin temporal rate at 3-hour and 12-hour flow (right). (c) Fluorescence intensity of phospho-Cofilin and mDia1 respect to flow time (3 independent experiments, n > 50 cells). (d) Schematic of endothelial cell subjected to unidirectional flow shear stress (USS) of 12 dyne cm^{-2} with decreasing phospho-Cofilin and mDia1 expression. Scale bars, 40μm. (e) The representative conventional fibers polarization of endothelial cells under USS of 12 dyne cm^{-2}. (f) Experimentally measured changes in nuclear height under unidirectional shear stress (3 independent experiments, n>80). Simulated nucleus z-direction deformation under unidirectional shear stress. (g) Correlation matrix analysis of the concentrations of cascade signaling pathways involved in the model, including RhoA, and the activated (inactivated) ROCK, myosin, LIMK, Cofilin, mDia, F-actin, and

YAP. Scale bars, 3μm. All data are presented as mean ± s.d. *$p<0.05$; **$p<0.01$; ***$p<0.001$; ****$p<0.0001$; NS, not significant.

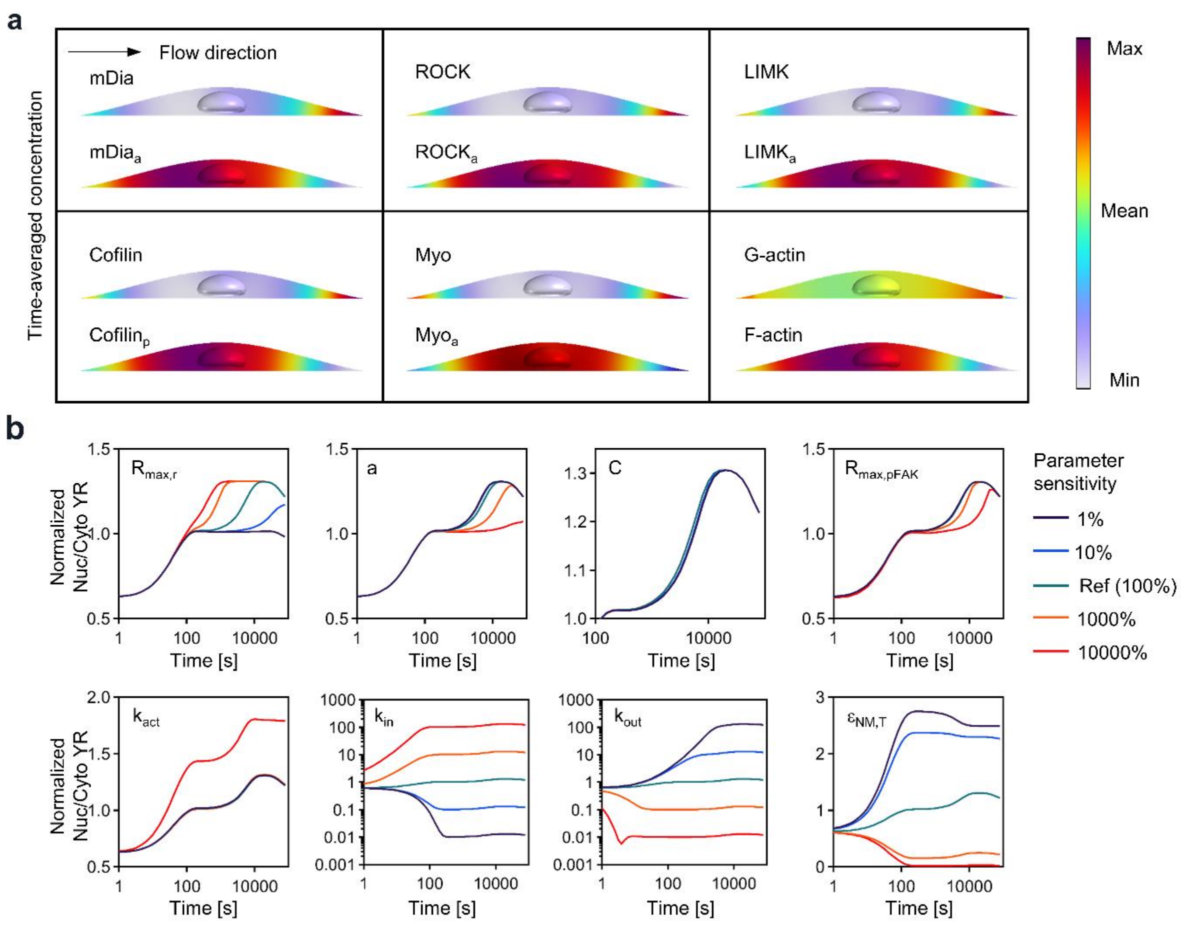

Figure S9. Computational signaling pathways and the sensitivity analysis. (a) Signaling pathways downstream of RhoA, including the mDia, ROCK, LIMK, Cofilin, myosin, and actin (time-averaged concentration among 24 hours' unidirectional flow of 12 dyne cm^{-2}). (b) Sensitivity analysis of key parameters involved in the mechanical process. Our analysis revealed that changes in the maximum activation rate of RhoA due to shear stress (Rmax,ρ), the shear stress value when the activation rate of RhoA reaches $R_{max,\rho}/2$ (a), the threshold von Mises stress at focal adhesion (C), the FAK phosphorylation rate ($R_{max,pFAK}$), and the activation stress due to myosin activation (k_{act}) do not significantly affect the biphasic translocation of YAP under unidirectional shear stress. However, alterations in YAP nuclear import rate (k_{in}), YAP nuclear export rate (k_{out}), and threshold nuclear membrane strain of YAP nuclear import ($\varepsilon_{NM,T}$) – which are directly related to the nuclear-cytoplasmic properties of YAP – have a more substantial impact on the YAP transport.

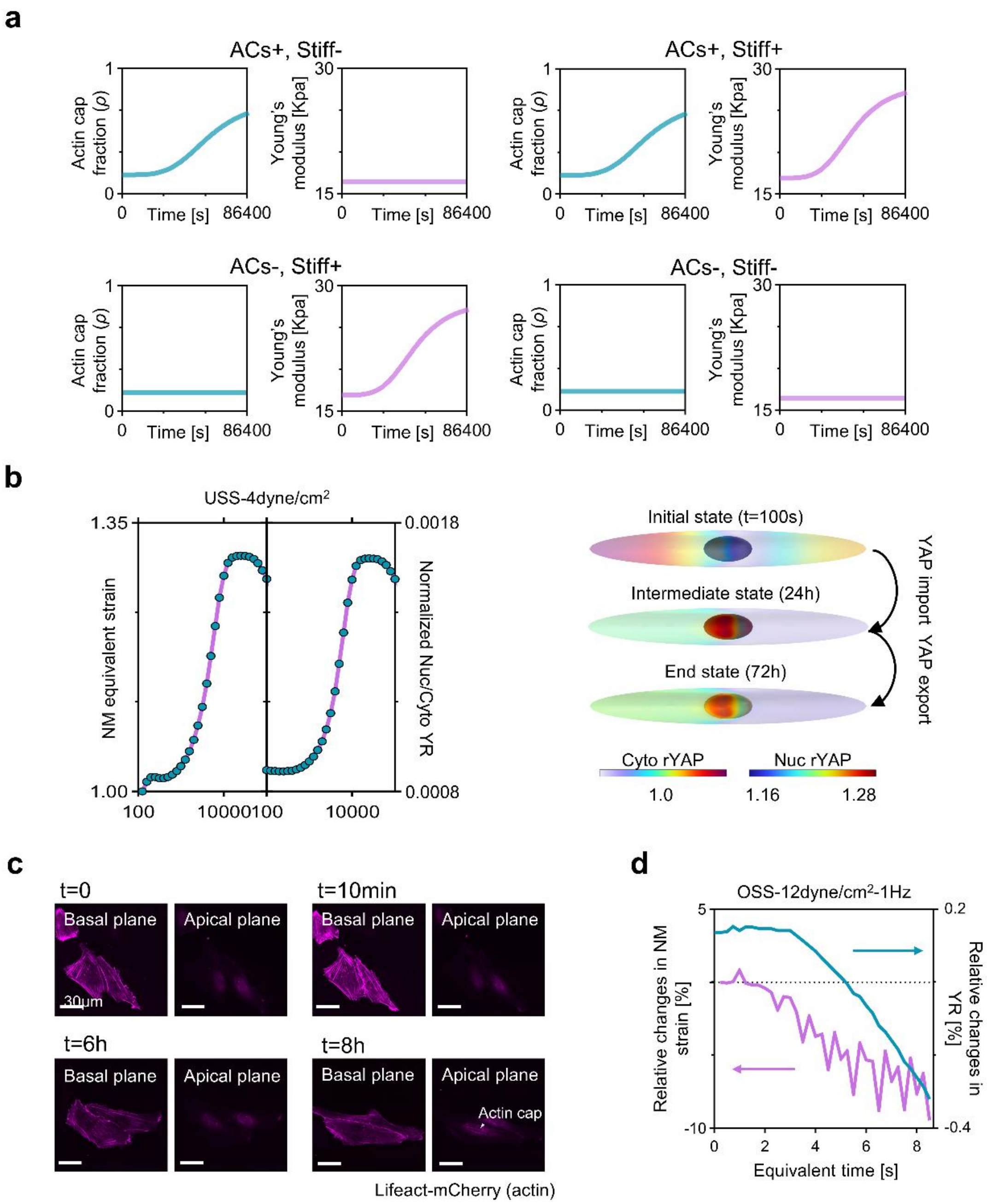

Figure S10. Supporting information of simulating the mechanotransduction process. (a) Simulation inputs for each condition including the actin cap formation (*ρ*) and the nuclear Young's modulus with respect to time. (b) Mechanochemical modeling under 4 dyne cm^{-2} unidirectional shear stress. (c) Live imaging of the actin cytoskeleton through Lifeact under 12 dyne/cm2, unidirectional flow. The results show a rapid increase in basal F-actin intensity (10 min), and followed by the gradual decrease. (d) The relative changes in NM strain and YR in OSS at 0±12 dyne cm^{-2}, 1Hz by enhancing actin cap formation and inhibiting nuclear softening.

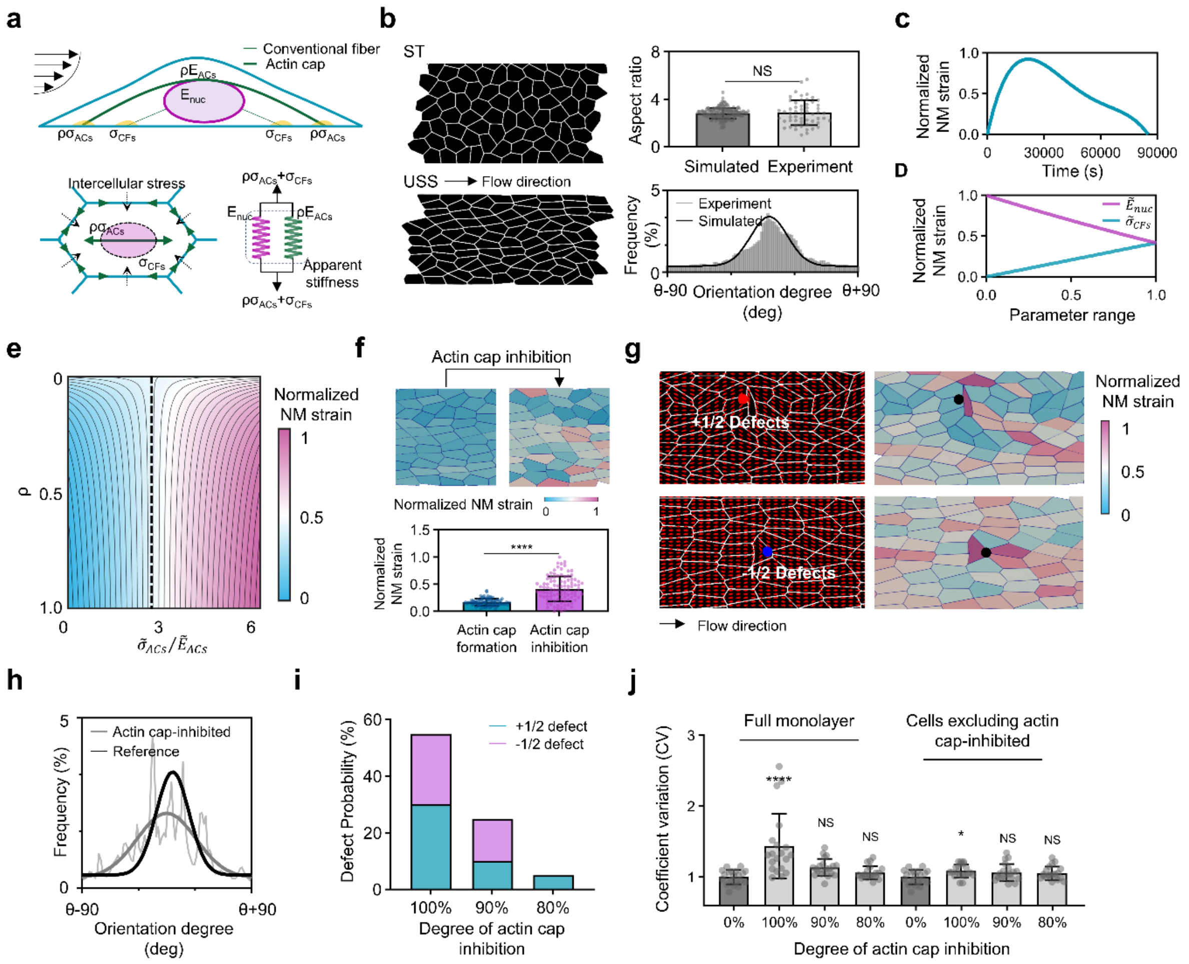

Figure S11. Regulatory role of actin cap in cell monolayer behavior and nuclear membrane strain. (a) Schematic of the vertex model considering actin cap and the minimal model to assess the nuclear membrane strain. (b) Static and USS-induced alignment, and elongation of endothelial cells. The comparison of experimental and simulated cell aspect ratio and polarization (the flow direction is denoted as θ). (c) Minimal model-calculated nuclear membrane strain over the duration of USS. (d) Dimensionless nuclear stiffness ($\tilde{E}_{nuc}$) and conventional fiber stress ($\tilde{\sigma}_{CFs}$) and their impact on nuclear membrane strain. (e) The influence of actin cap on nuclear membrane strain. (f) The effect of actin cap inhibition on collective cell nuclear membrane strain (n > 80, p < 0.0001, mean ± s.d.). (g) The inhibition of actin cap in individual cells and its potential role in inducing topological defects and spatially heterogeneous distribution of nuclear membrane strain (red dots represent +1/2 defects, blue dots represent -1/2 defects). (h) The effects of actin cap inhibition in individual cells on collective cell polarization. (i) The varying degrees of actin cap inhibition in individual cells impact the formation and probability of different defect types (n=20 in each condition). (j) The varying degrees of actin cap inhibition in individual cells influence the spatially heterogeneous distribution of nuclear membrane strain, including and excluding the

cells with inhibited actin cap (n=20 in each condition, ****$p < 0.0001$; *$p < 0.05$; NS, not significant).

Table S1. Equations of the mechanochemical model

<table>
<tr><td>1</td><td colspan="2">Fluid flow</td></tr>
<tr><td>1.1</td><td colspan="2">$\rho_{fluid}\left(\frac{\partial \mathbf{u_{fluid}}}{\partial t}+\mathbf{u_{fluid}}\cdot\nabla\mathbf{u_{fluid}}\right)=\mu\nabla^2\mathbf{u_{fluid}}-\nabla p$
$\nabla\cdot\mathbf{u_{fluid}}=0$</td></tr>
<tr><td>2</td><td colspan="2">Fluid solid interaction (FSI)</td></tr>
<tr><td>2.1</td><td colspan="2">$\mathbf{\Gamma}=-p\mathbf{I}+\mu\left(\nabla\mathbf{u_{fluid}}+\left(\nabla\mathbf{u_{fluid}}\right)^T\right)$
$\mathbf{\Gamma}\cdot\mathbf{n}=\boldsymbol{\sigma}\cdot\mathbf{n}$
$\mathbf{v_{wall}}=\frac{\partial\mathbf{u_{solid}}}{\partial t}$</td></tr>
<tr><td>3</td><td colspan="2">Solid mechanics</td></tr>
<tr><td>3.1</td><td>General form in all solid domain</td><td>$\rho_{solid}\frac{\partial^2\mathbf{u_{solid}}}{\partial t^2}=\nabla\cdot\boldsymbol{\sigma}$</td></tr>
<tr><td>3.2</td><td>Cytoplasm: isotropic linear elastic material</td><td>$\boldsymbol{\sigma}=\frac{E_{cyto}}{1+\nu}\boldsymbol{\varepsilon}+\frac{\nu E_{cyto}}{(1+\nu)(1-2\nu)}tr(\boldsymbol{\varepsilon})\mathbf{I}$</td></tr>
<tr><td>3.3</td><td>Cytoskeleton: along the fibers' direction (Fitting refers to the Hill fitting in Figure S7b and c; CFs refers to the conventional fibers)</td><td>$\sigma=\rho\sigma_i=\rho\left(k_F\varepsilon+\sigma_M\right)$
$\sigma_M=k_{act}C_{MyoA}$
$\rho=\begin{cases}Fitting\left(Figure.S7b\right), i=ActinCap\\ C_{F-actin}/C_{F-actin,\max}, i=CFs\end{cases}$</td></tr>
<tr><td>3.4</td><td>Nucleus: isotropic viscoelastic material (Maxwell model)</td><td>$\dot{\varepsilon}=\dot{\sigma}/E_{nuc}+\sigma/\tau_r$
$E_{nuc}=Fitting(Figure.S7c)$</td></tr>
<tr><td>4</td><td colspan="2">Transport model</td></tr>
<tr><td></td><td colspan="2">Module 1: Flow Shear Stress Sensing</td></tr>
<tr><td></td><td>Equations</td><td>Reaction Rate</td></tr>
<tr><td>4.1</td><td>$\frac{\partial C_{FAK}}{\partial t}=D_{FAK}\Delta C_{FAK}+R_1$
$-D_{FAK}\left(\mathbf{n}\cdot\nabla C_{FAK}\right)\big|_{PM-basal}=-T\cdot R_{pFAK}$</td><td rowspan="2">$R_1=k_{df}C_{pFAK}-k_fC_{FAK}$
$R_{pFAK}=R_{\max,pFAK}\frac{\sigma^2}{\sigma^2+C^2}C_{FAK}$</td></tr>
<tr><td>4.2</td><td>$\frac{\partial C_{pFAK}}{\partial t}=D_{pFAK}\Delta C_{pFAK}-R_1$
$-D_{pFAK}\left(\mathbf{n}\cdot\nabla C_{pFAK}\right)\big|_{PM-basal}=T\cdot R_{pFAK}$</td></tr>
<tr><td>4.3</td><td>$\frac{\partial C_{Rho}}{\partial t}=D_{Rho}\Delta C_{Rho}-R_2+R_3/n_r$</td><td>$R_2=k_{fk\rho}\left[\gamma C_{pFAK}{}^5+1\right]C_{Rho}$</td></tr>
</table>

	$-D_{Rho}\left(\mathbf{n}\cdot\nabla C_{Rho}\right)\big\|_{PM-apical}=-T\cdot R_{a\rho}$	$R_3=k_{d\rho}C_{RhoA}$
4.4	$\frac{\partial C_{RhoA}}{\partial t}=D_{RhoA}\Delta C_{RhoA}+n_r R_2-R_3$<br>$-D_{RhoA}\left(\mathbf{n}\cdot\nabla C_{RhoA}\right)\big\|_{PM-apical}=T\cdot R_{a\rho}$	$R_{a\rho}=R_{\max,\rho}\frac{\tau}{\tau+a}C_{Rho}$
	**Module 2: Cytoskeleton regulation**	
4.5	$\frac{\partial C_{ROCK}}{\partial t}=D_{ROCK}\Delta C_{ROCK}+R_4-R_5/n_r$	$R_4=k_{drock}C_{ROCK_A}$<br>$R_5=k_{r\rho}C_{ROCK}C_{RhoA}$
4.6	$\frac{\partial C_{ROCK_A}}{\partial t}=D_{ROCK_A}\Delta C_{ROCK_A}-R_4+R_5/n_r$	
4.7	$\frac{\partial C_{mDia}}{\partial t}=D_{mDia}\Delta C_{mDia}+R_6-R_7/n_r$	$R_6=k_{dmDia}C_{mDia_A}$<br>$R_7=k_{m\rho}C_{mDia}C_{RhoA}$
4.8	$\frac{\partial C_{mDia_A}}{\partial t}=D_{mDia_A}\Delta C_{mDia_A}-R_6+R_7/n_r$	
4.9	$\frac{\partial C_{Myo}}{\partial t}=D_{Myo}\Delta C_{Myo}-R_8$	$R_8=k_{mr}\left(\kappa C_{ROCK_A}+1\right)C_{Myo}$<br>$-k_{dmy}C_{MyoA}$
4.10	$\frac{\partial C_{MyoA}}{\partial t}=D_{MyoA}\Delta C_{MyoA}+R_8$	
4.11	$\frac{\partial C_{LIMK}}{\partial t}=D_{LIMK}\Delta C_{LIMK}-R_9$	$R_9=k_{lr}\left(\xi C_{ROCK_A}+1\right)C_{LIMK}$<br>$-k_{dl}C_{LIMK_A}$
4.12	$\frac{\partial C_{LIMK_A}}{\partial t}=D_{LIMK_A}\Delta C_{LIMK_A}+R_9$	
4.13	$\frac{\partial C_{Cofilin_p}}{\partial t}=D_{Cofilin_p}\Delta C_{Cofilin_p}-R_{10}$	$R_{10}=k_{turn-over}C_{Cofilin_p}-$<br>$\frac{k_{catCofilin}C_{LIMK_A}C_{Cofilin_{np}}}{k_{mCofilin}+C_{Cofilin_{np}}}$
4.14	$\frac{\partial C_{Cofilin_{np}}}{\partial t}=D_{Cofilin_{np}}\Delta C_{Cofilin_{np}}+R_{10}$	
4.15	$\frac{\partial C_{G-actin}}{\partial t}=D_{G-actin}\Delta C_{G-actin}-R_{11}$	$R_{11}=k_{ra}\left(\lambda C_{mDia_A}+1\right)C_{G-actin}$<br>$-\left(k_{dep}+k_{fc1}C_{Cofilin_{np}}\right)C_{F-actin}$
4.16	$\frac{\partial C_{F-actin}}{\partial t}=D_{F-actin}\Delta C_{F-actin}+R_{11}$	
	**Module 3: YAP nucleocytoplasmic transport**	
4.17	$\frac{\partial C_{pYAP}}{\partial t}=D_{pYAP}\Delta C_{pYAP}-R_{12}$	$R_{12}=\left(k_{CN}+k_{CY}C_{F-actin}C_{MyoA}\right)C_{pYAP}$<br>$-k_{NC}C_{YAP}$<br>$J_{YAP,NM}=k_{in}\frac{\varepsilon_{NM}}{\varepsilon_{NM}+\varepsilon_{NM,T}}C_{YAP}-k_{out}C_{YAP_{nuc}}$
4.18	$\frac{\partial C_{YAP}}{\partial t}=D_{YAP}\Delta C_{YAP}+R_{12}-J_{YAP,NM}$	
4.19	$\frac{\partial C_{YAP_{nuc}}}{\partial t}=D_{YAP_{nuc}}\Delta C_{YAP_{nuc}}+J_{YAP,NM}$	

Table S2. Parameters in the mechanochemical model

Parameter	Value	Description
ρ_{fluid}	1000kg m^{-3}	Density of the fluid
μ	0.95×10^{-3}pa·s	Viscosity of the fluid
ρ_{cyto}	1060kg m^{-3}	Density of the cytoplasm
E_{cyto}	2.9kPa	Young's modulus of the cytoplasm[25]
ν	0.45	Poisson ratio of the nucleus and cytoplasm[26]
ρ_{nuc}	1350kg m^{-3}	Density of the nucleus, estimated from[27]
E_{nuc}	Fitted from Brillouin measurement (see Figure S7c)	Young's modulus of the nucleus, which is a function of flow time in each shear stress condition
τ_r	0.3s	Relaxation time of the nucleus[6]
ρ	Fitted from actin cap present cells (see Figure S7b)	Volume fraction of actin fibers
$R_{max,pFAK}$	0.379s^{-1}	FAK phosphorylation rate due to stress at focal adhesion applied by actin filaments[28]
k_f	0.015s^{-1}	Baseline phosphorylation rate of FAK[28]
k_{df}	0.035s^{-1}	Deactivation rate of FAK[4b, 29]
C	110Pa	Threshold von mises stress at focal adhesion, estimated based on[30]
k_{fkp}	1s^{-1}	Activation rate of RhoA at focal adhesion due to pFAK[4b]
k_{dp}	0.625s^{-1}	Deactivation rate of RhoA[31]
γ	1000μM^{-5}	Activation rate of RhoA due to pFAK[4a]
$R_{max,\rho}$	1.32s^{-1}	Maximum activation rate of RhoA due to the shear stress, which is selected to close the order of magnitude for RhoA activation rate due to pFAK[4a]
a	0.5Pa	Threshold shear stress at cell membrane[32]
n_r	5	Scale for converting volume and surface RhoA[4a], estimated by matching staining results in Figure 3A
k_{drock}	0.8s^{-1}	Deactivation rate of ROCK[4b, 33]

k_{rp}	$0.648s^{-1}\mu M^{-1}$	Activation rate of ROCK due to RhoA[34]
k_{dmdia}	$0.005s^{-1}$	Deactivation rate of mDia[35]
k_{mr}	$0.03s^{-1}$	Activation rate of mDia due to RhoA[34]
κ	$36\mu M^{-1}$	Amplification of myosin to ROCK[34]
k_{dmy}	$0.067s^{-1}$	Deactivation rate of activated myosin[4]
K_F	1.45MPa	Young's modulus of stress fiber[36]
k_{act}	$1000J\ mol^{-1}$	Activate stress due to myosin activation, estimated by matching the magnitude order of passive stress in actin fibers
k_{lr}	$0.07s^{-1}$	Activation rate of LIMK[4]
ξ	$55.49\mu M^{-1}$	Amplification of LIMK to ROCK[4a]
k_{dl}	$2s^{-1}$	Deactivation rate of $LIMK_A$[4]
$k_{turn\text{-}over}$	$0.04s^{-1}$	Activation rate of cofilin[4]
$k_{catCofilin}$	$0.34s^{-1}$	Rate of catalysis for cofilin phosphorylation[37]
$k_{mcofilin}$	$4\mu M$	Concentration of cofilin when the reaction rate reaches its half of max rate[37]
k_{ra}	$0.4s^{-1}$	Activation (polymerization) rate of F-actin[4]
λ	$50\mu M^{-1}$	Amplification of F-actin to mDia[4]
k_{dep}	$3.5s^{-1}$	Deactivation (depolymerization) rate of F-actin[4]
k_{fc1}	$4s^{-1}\mu M^{-1}$	Deactivation (depolymerization) rate of F-actin due to cofilin[4]
k_{CN}	$0.56s^{-1}$	Baseline rate of YAP dephosphorylation[4a, 12b, 38]
k_{CY}	$7.6\times10^{-4}\mu M^{-1}s^{-1}$	Rate of YAP dephosphorylation due to stress fiber[4a, 12b, 38]
$\varepsilon_{NM,T}$	0.0002	Threshold first principal strain of YAP nuclear import, estimated by matching the magnitude order of the calculated strain in pure mechanical model
k_{NC}	$0.14s^{-1}$	Rate of YAP phosphorylation[4a, 12b, 38]
k_{in}	$10s^{-1}\mu M^{-1}$	Maximum rate of YAP nuclear import[4a, 12b, 38]
k_{out}	$10s^{-1}\mu M^{-1}$	Nuclear export rate of YAP[12a]

References

[1] a) F. Alisafaei, D. S. Jokhun, G. Shivashankar, V. B. Shenoy, *Proceedings of the National Academy of Sciences* **2019**, *116* (27), 13200; b) E. McEvoy, T. Sneh, E. Moeendarbary, Y. Javanmardi, N. Efimova, C. Yang, G. E. Marino-Bravante, X. Chen, J. Escribano, F. Spill, *Nature communications* **2022**, *13* (1), 7089.
[2] J. K. Novev, M. L. Heltberg, M. H. Jensen, A. Doostmohammadi, *Integrative Biology* **2021**, *13* (8), 197, https://doi.org/10.1093/intbio/zyab012.
[3] A. Ahmad Bakir, A. Al Abed, M. C. Stevens, N. H. Lovell, S. Dokos, *Frontiers in physiology* **2018**, *9*, 1259.
[4] a) K. E. Scott, S. I. Fraley, P. Rangamani, *Proceedings of the National Academy of Sciences* **2021**, *118* (20), e2021571118; b) M. Sun, F. Spill, M. H. Zaman, *Biophysical journal* **2016**, *110* (11), 2540.
[5] A. B. Chambliss, S. B. Khatau, N. Erdenberger, D. K. Robinson, D. Hodzic, G. D. Longmore, D. Wirtz, *Scientific reports* **2013**, *3* (1), 1.
[6] a) O. Wintner, N. Hirsch‐Attas, M. Schlossberg, F. Brofman, R. Friedman, M. Kupervaser, D. Kitsberg, A. Buxboim, *Advanced Science* **2020**, *7* (8), 1901222; b) J. Swift, I. L. Ivanovska, A. Buxboim, T. Harada, P. D. P. Dingal, J. Pinter, J. D. Pajerowski, K. R. Spinler, J.-W. Shin, M. Tewari, *Science* **2013**, *341* (6149), 1240104.
[7] S. Tiveci, A. Akın, T. Çakır, H. Saybaşılı, K. Ülgen, *Computational biology and chemistry* **2005**, *29* (2), 151.
[8] G. Hong, J. Z. Wu, J. T. Robinson, H. Wang, B. Zhang, H. Dai, *Nature communications* **2012**, *3* (1), 700.
[9] a) S. R. Hoare, P. H. Tewson, A. M. Quinn, T. E. Hughes, L. J. Bridge, *Scientific reports* **2020**, *10* (1), 1; b) M. J. Paszek, D. Boettiger, V. M. Weaver, D. A. Hammer, *PLoS computational biology* **2009**, *5* (12), e1000604.
[10] D.-H. Kim, S. B. Khatau, Y. Feng, S. Walcott, S. X. Sun, G. D. Longmore, D. Wirtz, *Scientific reports* **2012**, *2* (1), 555.
[11] a) H. Nakajima, K. Yamamoto, S. Agarwala, K. Terai, H. Fukui, S. Fukuhara, K. Ando, T. Miyazaki, Y. Yokota, E. Schmelzer, *Developmental cell* **2017**, *40* (6), 523; b) S. Dupont, L. Morsut, M. Aragona, E. Enzo, S. Giulitti, M. Cordenonsi, F. Zanconato, J. Le Digabel, M. Forcato, S. Bicciato, *Nature* **2011**, *474* (7350), 179; c) K.-C. Wang, Y.-T. Yeh, P. Nguyen, E. Limqueco, J. Lopez, S. Thorossian, K.-L. Guan, Y.-S. J. Li, S. Chien, *Proceedings of the National Academy of Sciences* **2016**, *113* (41), 11525; d) L. Wang, J.-Y. Luo, B. Li, X. Y. Tian, L.-J. Chen, Y. Huang, J. Liu, D. Deng, C. W. Lau, S. Wan, *Nature* **2016**, *540* (7634), 579.
[12] a) I. Andreu, I. Granero-Moya, N. R. Chahare, K. Clein, M. Molina-Jordán, A. E. Beedle, A. Elosegui-Artola, J. F. Abenza, L. Rossetti, X. Trepat, *Nature Cell Biology* **2022**, 1; b) A. Elosegui-Artola, I. Andreu, A. E. Beedle, A. Lezamiz, M. Uroz, A. J. Kosmalska, R. Oria, J. Z. Kechagia, P. Rico-Lastres, A.-L. Le Roux, *Cell* **2017**, *171* (6), 1397.
[13] a) T. Nagai, H. Honda, *Philosophical Magazine B* **2001**, *81* (7), 699; b) A. G. Fletcher, J. M. Osborne, P. K. Maini, D. J. Gavaghan, *Progress in biophysics and molecular biology* **2013**, *113* (2), 299.
[14] D. Bi, X. Yang, M. C. Marchetti, M. L. Manning, *Physical Review X* **2016**, *6* (2), 021011.
[15] S. Tlili, J. Yin, J.-F. Rupprecht, M. Mendieta-Serrano, G. Weissbart, N. Verma, X. Teng, Y. Toyama, J. Prost, T. Saunders, *Proceedings of the National Academy of Sciences* **2019**, *116* (51), 25430.
[16] S.-Z. Lin, M. Merkel, J.-F. Rupprecht, *The European Physical Journal E* **2022**, *45* (1), 4.

[17] a) S. Sonam, L. Balasubramaniam, S.-Z. Lin, Y. M. Y. Ivan, I. Pi-Jaumà, C. Jebane, M. Karnat, Y. Toyama, P. Marcq, J. Prost, *Nature Physics* **2023**, *19* (1), 132; b) S. J. DeCamp, G. S. Redner, A. Baskaran, M. F. Hagan, Z. Dogic, *Nature materials* **2015**, *14* (11), 1110.
[18] a) G. R. Mirams, C. J. Arthurs, M. O. Bernabeu, R. Bordas, J. Cooper, A. Corrias, Y. Davit, S.-J. Dunn, A. G. Fletcher, D. G. Harvey, *PLoS computational biology* **2013**, *9* (3), e1002970; b) F. R. Cooper, R. E. Baker, M. O. Bernabeu, R. Bordas, L. Bowler, A. Bueno-Orovio, H. M. Byrne, V. Carapella, L. Cardone-Noott, J. Cooper, *Journal of Open Source Software* **2020**.
[19] a) T. B. Saw, A. Doostmohammadi, V. Nier, L. Kocgozlu, S. Thampi, Y. Toyama, P. Marcq, C. T. Lim, J. M. Yeomans, B. Ladoux, *Nature* **2017**, *544* (7649), 212; b) K. D. Endresen, M. Kim, M. Pittman, Y. Chen, F. Serra, *Soft Matter* **2021**, *17* (24), 5878.
[20] a) B. Ladoux, R.-M. Mège, *Nature reviews Molecular cell biology* **2017**, *18* (12), 743; b) S. Jain, V. M. Cachoux, G. H. Narayana, S. de Beco, J. D'alessandro, V. Cellerin, T. Chen, M. L. Heuzé, P. Marcq, R.-M. Mège, *Nature physics* **2020**, *16* (7), 802.
[21] S. Alt, P. Ganguly, G. Salbreux, *Philosophical Transactions of the Royal Society B: Biological Sciences* **2017**, *372* (1720), 20150520.
[22] Q. Shi, L. Zheng, J. Na, X. Li, Z. Yang, X. Chen, Y. Song, C. Li, L. Zhou, Y. Fan, *Cellular and Molecular Life Sciences* **2022**, *79* (11), 1.
[23] J.-Y. Shiu, L. Aires, Z. Lin, V. Vogel, *Nature cell biology* **2018**, *20* (3), 262.
[24] N. Koushki, A. Ghagre, L. K. Srivastava, C. Molter, A. J. Ehrlicher, *Proceedings of the National Academy of Sciences* **2023**, *120* (28), e2301285120.
[25] A. J. Engler, M. A. Griffin, S. Sen, C. G. Bonnemann, H. L. Sweeney, D. E. Discher, *The Journal of cell biology* **2004**, *166* (6), 877.
[26] T. Abdalrahman, N. H. Davies, T. Franz, *Medical & Biological Engineering & Computing* **2021**, *59* (9), 1933.
[27] K. Kim, J. Guck, *Biophysical Journal* **2020**, *119* (10), 1946.
[28] J. A. Beamish, E. Chen, A. J. Putnam, *PLoS One* **2017**, *12* (7), e0181085.
[29] S. Kumar, A. Das, S. Sen, *Molecular bioSystems* **2014**, *10* (4), 838.
[30] J. Stricker, Y. Beckham, M. W. Davidson, M. L. Gardel, *PloS one* **2013**, *8* (7), e70652.
[31] Y. Sako, K. Hibino, T. Miyauchi, Y. Miyamoto, M. Ueda, T. Yanagida, *Single Molecules* **2000**, *1* (2), 159.
[32] Q. Wan, S. J. Kim, H. Yokota, S. Na, *Cell biology international* **2013**, *37* (6), 568.
[33] J. Feng, M. Ito, Y. Kureishi, K. Ichikawa, M. Amano, N. Isaka, K. Okawa, A. Iwamatsu, K. Kaibuchi, D. J. Hartshorne, *Journal of Biological Chemistry* **1999**, *274* (6), 3744.
[34] M. C. Lampi, C. J. Faber, J. Huynh, F. Bordeleau, M. R. Zanotelli, C. A. Reinhart-King, *PLoS One* **2016**, *11* (1), e0147033.
[35] L. Cao, M. Kerleau, E. L. Suzuki, H. Wioland, S. Jouet, B. Guichard, M. Lenz, G. Romet-Lemonne, A. Jegou, *Elife* **2018**, *7*, e34176.
[36] J. S. Milner, M. W. Grol, K. L. Beaucage, S. J. Dixon, D. W. Holdsworth, *Journal of functional biomaterials* **2012**, *3* (1), 209.
[37] P. Rangamani, M. G. Levy, S. Khan, G. Oster, *Proceedings of the National Academy of Sciences* **2016**, *113* (36), E5298.
[38] J. Y. Lee, J. K. Chang, A. A. Dominguez, H.-p. Lee, S. Nam, J. Chang, S. Varma, L. S. Qi, R. B. West, O. Chaudhuri, *Nature communications* **2019**, *10* (1), 1.
